# Metabolomic Analysis of Diverse Mice Reveals Hepatic Arginase-1 as Source of Plasma Arginase in Plasmodium chabaudi Infection

**DOI:** 10.1128/mBio.02424-21

**Published:** 2021-10-05

**Authors:** Nicole M. Davis, Michelle M. Lissner, Crystal L. Richards, Victoria Chevée, Avni S. Gupta, Frank C. Gherardini, David S. Schneider

**Affiliations:** a Department of Microbiology and Immunology, Stanford University, Stanford, California, USA; b Laboratory of Bacteriology, National Institutes of Health, National Institute of Allergy and Infectious Diseases, Rocky Mountain Laboratories, Hamilton, Montana, USA; Columbia University Medical Center; University of California, Irvine

**Keywords:** malaria, *Plasmodium chabaudi*, metabolomics, disease severity, arginine, *Plasmodium*, malaria

## Abstract

Infections disrupt host metabolism, but the factors that dictate the nature and magnitude of metabolic change are incompletely characterized. To determine how host metabolism changes in relation to disease severity in murine malaria, we performed plasma metabolomics on eight Plasmodium chabaudi-infected mouse strains with diverse disease phenotypes. We identified plasma metabolic biomarkers for both the nature and severity of different malarial pathologies. A subset of metabolic changes, including plasma arginine depletion, match the plasma metabolomes of human malaria patients, suggesting new connections between pathology and metabolism in human malaria. In our malarial mice, liver damage, which releases hepatic arginase-1 (Arg1) into circulation, correlated with plasma arginine depletion. We confirmed that hepatic Arg1 was the primary source of increased plasma arginase activity in our model, which motivates further investigation of liver damage in human malaria patients. More broadly, our approach shows how leveraging phenotypic diversity can identify and validate relationships between metabolism and the pathophysiology of infectious disease.

## INTRODUCTION

Malaria is a serious infectious disease caused by Apicomplexan parasites in the genus *Plasmodium.* The most virulent species in humans is Plasmodium falciparum, which killed 405,000 people in 2018 according to the World Health Organization (https://www.who.int/news-room/fact-sheets/detail/malaria). Malarial pathologies range from uncomplicated fever and anemia to severe and sometimes fatal conditions, including severe anemia, metabolic acidosis, acute kidney injury, multiorgan failure, respiratory distress, and cerebral malaria (1–5).

Host metabolic changes frequently accompany malaria, often in association with severe systemic disease or organ-specific pathologies. For example, hepatocellular injury leads to elevated plasma levels of the metabolic enzymes aspartate and alanine transaminase (AST and ALT) (6, 7). Kidney dysfunction also leads to plasma metabolic changes, the most characteristic being elevated plasma urea and creatinine (8, 9). Dysfunction in either organ can lead to organ failure and other systemic complications (7). Hypoglycemia (10), metabolic acidosis (5), and other metabolic changes also accompany severe disease. However, it remains largely unclear how metabolic changes relate to the nature and degree of malaria pathophysiology.

Metabolites have recently emerged as important controllers of infection pathophysiology. Manipulation of glycolysis, for example, alters disease severity in malaria (11, 12) and controls tissue damage in other infections (13). Other metabolic targets, including iron (14), immunomodulatory metabolic enzymes like arginase (15–17), and metabolic hormones (18) have high potential to reduce pathology during infection. Undoubtedly, many metabolic therapies have yet to be discovered.

To uncover metabolic processes that are important during *Plasmodium* infection, we recently identified hundreds of plasma metabolites that change significantly when C57BL/6 mice are infected with Plasmodium chabaudi (19). Many of these metabolites also change in human malaria (4, 5, 19–22), but the causes and consequences of many of these metabolic changes remain unknown.

**FIG 1:**
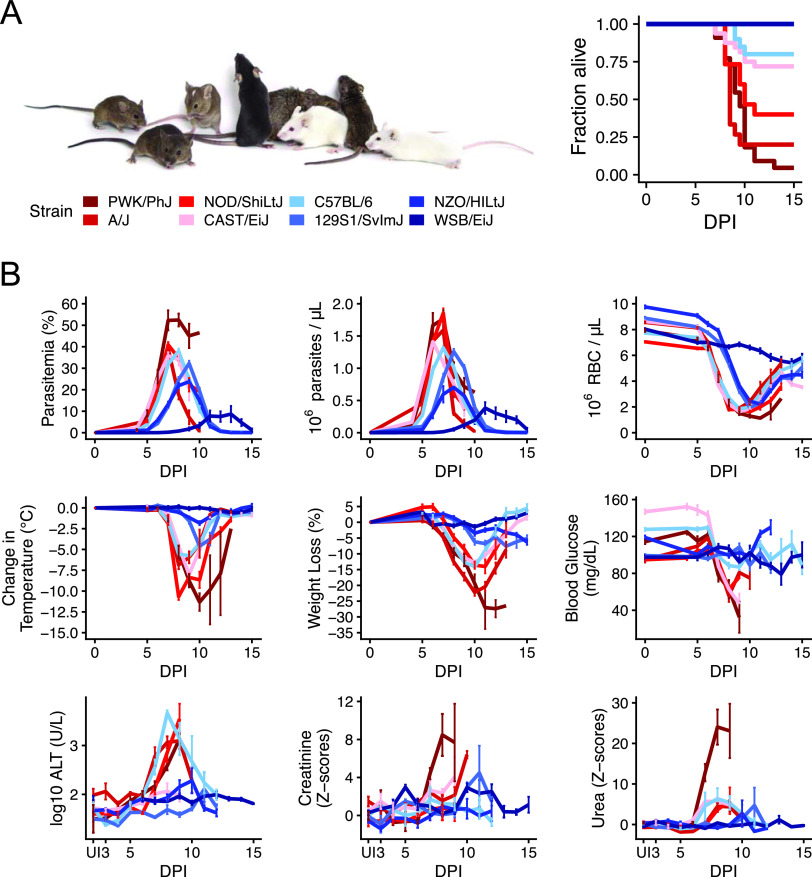
Malarial disease severity varies widely across eight genetically diverse mouse strains. (A) The eight founder strains (left to right): CAST/EiJ, 129S1/SvImJ, WSB/EiJ, C57BL/6J, NZO/HILtJ, NOD/ShiLtJ, PWK/PhJ, and A/J. For survival, strain colors reflect average survival and parasite load, with dark blue for the most resilient/fewest parasites and dark red for the least resilient (*n* = 10 for 100% resilient WSB/EiJ, NZO/HILtJ, and 129S1/SvImJ; *n* = 30 for 73% resilient C57BL/6; *n* = 32 for 72% resilient CAST/EiJ; *n* = 15 for 40% resilient NOD/ShiLtJ; *n* = 15 for 20% resilient A/J; *n* = 22 for 4.5% resilient PWK/PhJ). DPI, days postinfection. Photo reproduced with permission from Jackson Laboratories. (B) Nine metrics of disease severity across the eight founder strains over the course of acute P. chabaudi infection. Parasitemia = percent parasitized RBCs, parasite density = 10^6^ RBCs per microliter blood, anemia = 10^6^ RBCs per microliter blood). Lines indicate the mean (± SE) of ≥5 mice per strain per day. Z-scores = standard deviations from the means for uninfected C57BL/6 mice.

In a typical experiment, the field uses inbred strains to limit signal to noise to increase our probability of finding significant results. Here we used a collection of diverse inbred strains to deliberately introduce variation and then measure that variation. We selected eight inbred mouse lines (C57BL/6, WSB/EiJ, NZO/HILtJ, 129S1/SvImJ, A/J, CAST/EiJ, PWK/PhJ, and NOD/ShiLtJ) that represent 90% of laboratory mouse genetic diversity (23) and vary widely in P. chabaudi infection severity as measured by survival, anemia, parasite load, temperature loss, and weight loss (Fig. 1; see also Fig. S1 in the supplemental material). These founder strains were used to establish the Collaborative Cross (CC) and Diversity Outbred (DO) mouse populations (24), which more closely approximate human genetic and phenotypic diversity than individual inbred lines. Collectively, these groups of diverse mice have improved our understanding of genetic and immune factors that alter the severity of infections like influenza and tuberculosis (25–30). Our goal was to use diverse mice to identify metabolic factors that alter infection severity in malaria.

10.1128/mBio.02424-21.4 FIG S1 Malarial disease severity varies widely across eight genetically diverse mouse strains. Individual data points for plots shown in Fig. 1B (*n* = 3 to 5 mice per strain per day). Lines indicate mean values. Parasitemia = percent parasitized RBCs, parasite density = millions of RBCs per microliter of blood, RBC = millions of RBCs per microliter blood, temperature = change in body temperature (Celsius), body weight = change in body weight (grams), blood glucose = milligrams/deciliter, ALT = log_10_ U/liter, urea and creatinine = scaled imputed ion counts. Download FIG S1, EPS file, 1.8 MB. Copyright © 2021 Davis et al. 2021 Davis et al. https://creativecommons.org/licenses/by/4.0/ This content is distributed under the terms of the Creative Commons Attribution 4.0 International license.

To link malaria pathophysiology with host metabolic changes, we measured plasma metabolites and markers of pathology and immune responses in the eight P. chabaudi-infected founder strains. Using principal component analysis, we found that the magnitude of metabolic response to P. chabaudi infection was directly related to disease severity. Using correlation analysis, we found that arginine depletion was a specific marker for malaria-induced liver damage. We identified additional candidate molecules for future study. We showed that P. chabaudi-induced liver damage releases arginine-consuming hepatic arginase-1 (Arg1) into circulation, which explains increased plasma arginase activity in our model and may explain elevated plasma arginase in human malaria. Our results support the use of diverse mice to understand the links between metabolism and disease severity during infection and motivate further investigation of the metabolic consequences of liver damage in human malaria.

## RESULTS

### Infection alters metabolomic profiles in diverse mice.

**FIG 2:**
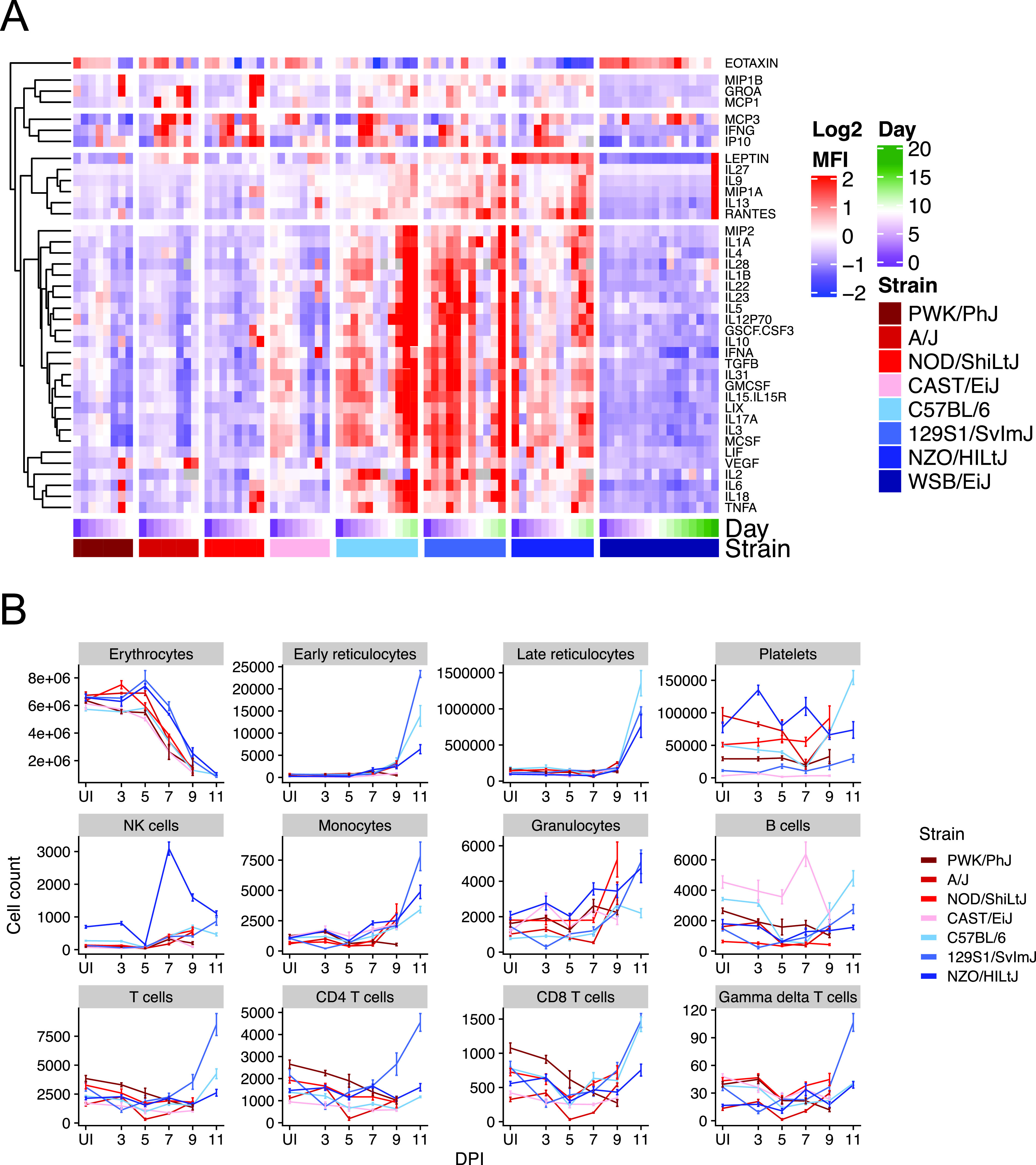
Immune responses to malaria vary widely across eight genetically diverse mouse strains. (A) Values of 38 hierarchically clustered cytokines (rows). Each column shows the median values for each of eight P. chabaudi-infected mouse strains on each day of infection (day 0 and days 3 to 9 for A/J, CAST/EiJ, NOD/ShiLtJ, and PWK/PhJ; days 0 and 3 to 11 for NZO/HILtJ; days 0 and 3 to 12 for C57BL/6 and 129S1/SvImJ; days 0 and 3 to 17 for WSB/EiJ). Each block of columns corresponds to a mouse strain, shown from least resilient (left) to most resilient (right). Within each column block, cells are ordered by day of infection (purple to green, white = day 9). Cell color reflects the median of Z-scored, raw median fluorescence intensity. Gray cells indicate missing data. (B) Whole-blood flow cytometry for 12 cell types are shown for seven of eight founder strains. Values for WSB/EiJ mice were omitted because the TER119 (erythroid lineage) marker failed to mark WSB/EiJ cells (*n* = 3 or 4 mice per strain per day). The mean of absolute cell counts (± standard error) are shown. Note that counting beads were not added to A/J samples; bead counts were estimated using the mean of bead counts from the experiment performed immediately after the A/J experiment.

We previously identified 370 metabolites that change significantly during P. chabaudi infection in C57BL/6 mice (19). However, wild-type C57BL/6 mice are largely resilient to infection, which limited our ability to understand how host metabolism changes in severe disease. To measure metabolism in mice with a broader range of disease severities, we used the eight founder strains (C57BL/6, WSB/EiJ, NZO/HILtJ, 129S1/SvImJ, A/J, CAST/EiJ, PWK/PhJ, and NOD/ShiLtJ), which vary widely in survival following P. chabaudi infection (Fig. 1A). For each strain, we monitored disease severity, plasma metabolites, plasma cytokines, and circulating immune cells during acute infection (days 5 to 12 postinfection or days 5 to 17 for WSB/EiJ mice, *n* = 5 infected mice per strain per day), when metabolic perturbations in C57BL/6 mice are greatest (19). The founder strains varied widely across nine metrics of malaria severity that largely tracked with survival and parasite load: anemia (red blood cells [RBCs] per microliter of blood), hypothermia, weight loss, hypoglycemia, liver injury (plasma ALT), and kidney injury (plasma urea, creatinine) (Fig. 1B and Fig. S1). Plasma cytokines tended to be higher in resilient strains than in nonresilient strains, with two exceptions: cytokines in resilient WSB/EiJ mice remained low throughout infection (Fig. 2A), and two nonresilient strains (PWK/PhJ and NOD/ShiLtJ) mounted hyperinflammatory responses (e.g., high gamma interferon [IFN-γ], tumor necrosis factor alpha [TNF-α], and interleukin 6 [IL-6] and low IL-10 and transforming growth factor β [TGF-β]) on day 8 or 9. Immune cell responses were largely consistent across strains, with elevated T cells, B cells, NK cells, and monocytes between days 7 to 9 postinfection (Fig. 2B). NK cells were particularly high in resilient NZO/HILtJ mice, as were T and B cells in resilient 129S1/SvImJ and nonresilient CAST/EiJ mice, respectively. Collectively, our data highlight the diversity of disease and immune phenotypes in the eight founder strains.

Interstrain variation in malarial disease severity allowed us to ask several questions about the relationship between disease severity and metabolism: is overall metabolic disruption greater in sicker animals? How do individual metabolites relate to different facets of disease? Can these metabolic markers of pathology provide new mechanistic insights into the pathophysiology of malaria or other infectious diseases?

**FIG 3:**
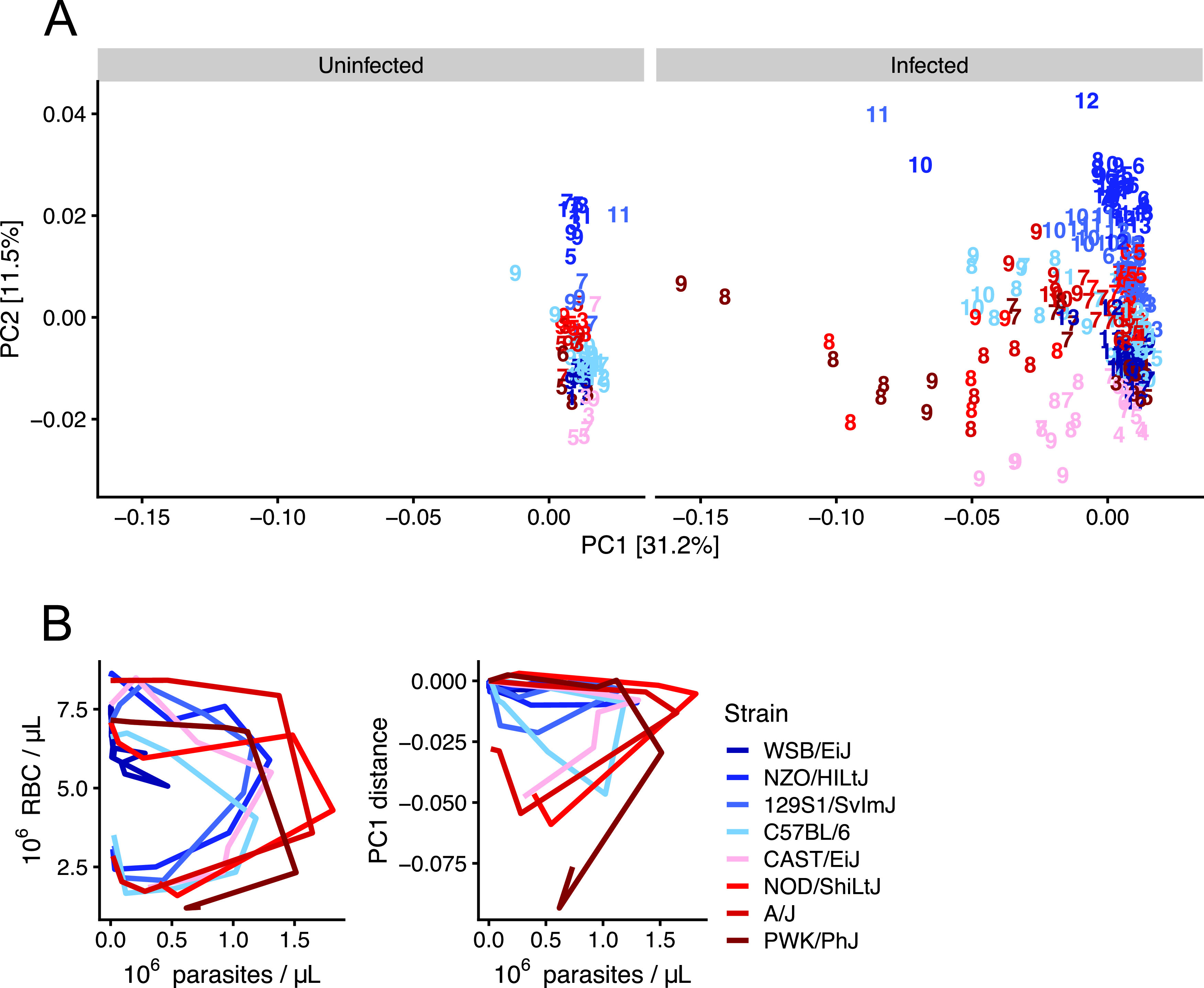
Genetic background and *Plasmodium* infection influence murine plasma metabolomes. (A) Bray-Curtis dissimilarity index was computed on 635 metabolites per sample for infected and uninfected samples. Principal component analysis (PCA) was performed, and sample positions in the first two principal components are displayed. PC1 and PC2 explain 31.2% and 11.5% variation, respectively. Each mouse sample is represented as a number that indicates days postinfection (or mock infection). (B) Disease space loops of RBCs and median PC1 values (derived from Fig. 2 data) plotted against median parasite densities throughout acute P. chabaudi infection. Each line represents the median value for ≥5 mice per strain per day (except final days for nonresilient strains in which <5 individuals remain).

To answer how disease severity impacted the overall composition of plasma metabolomes, we performed dimensionality reduction and visualization of 635 metabolites (see Materials and Methods) in the same samples used to determine disease severity using principal component analysis (PCA). Variance in principal component 2 (PC2) (11.2%) was associated with baseline differences among mouse strains, with NZO/HILtJ samples having the highest PC2 values and wild-derived strains (CAST/EiJ, WSB/EiJ, and PWK/PhJ) having the lowest PC2 values (Fig. 3A). Variance in PC1 (31.2%) was associated with infection severity. Highly resilient mice (WSB/EiJ, NZO/HILtJ, and 129S1/SvImJ) had high PC1 values throughout infection, while nonresilient strains had low PC1 values during periods of acute illness (days 7 to 10). Phase curves of PC1 versus parasite density show larger loops in disease space for less resilient strains, as is expected for variables that influence disease severity (Fig. 3B) (31). We next used canonical correspondence analysis (CCA), a technique similar to PCA, to identify molecules that drove the most variation among samples (see Table S1 in the supplemental material). Molecules that differentiated sick from healthy samples were associated with altered feeding behavior (food components, ketosis markers, indoles, serotonin, and acylcarnitines), oxidative stress (glutathione and 2-hydroxyisobutyrate), and liver and kidney dysfunction (formiminoglutamate, bilirubin, homocitrulline, urea, *p*-cresol glucuronide, *p*-cresol sulfate, and *N*-acetyltyrosine). We also performed Student’s *t* tests to identify molecules that differentiate resilient and nonresilient mice before infection (Table S2). Plasma levels of a number of metabolites differed significantly between uninfected resilient and nonresilient mice, including the food component and osmolyte betaine (32), sphingomyelins, lysine, and pantothenic acid.

10.1128/mBio.02424-21.1 TABLE S1 Metabolites whose CCA vectors indicate acute infection in malaria. Infected and uninfected samples from all days and mouse strains were included in the analysis. Scaled imputed ion counts were Z-scored using uninfected C57BL/6 values as the mean. A pseudocount was added to all values to make the minimum value = 1 (Bray-Curtis dissimilarity cannot be computed on negative values). Only metabolites present in ≥80% of samples were included in analysis, and bile acids were removed from analysis prior to correlations because many were inconsistently detected across strains. For CCA, any metabolite with a vector length of > 0.1 in CA1 or CA2 was included in the comparison to identify metabolites that discriminate between health and disease. Download Table S1, DOCX file, 0.05 MB. Copyright © 2021 Davis et al. 2021 Davis et al. https://creativecommons.org/licenses/by/4.0/ This content is distributed under the terms of the Creative Commons Attribution 4.0 International license.

10.1128/mBio.02424-21.2 TABLE S2 Student’s *t* test results. *t* tests were computed to compare the mean values of metabolites in healthy mice from mouse strains that are resilient (WSB/EiJ, NZO/HILtJ, 129S1/SvImJ, and C57BL/6) and nonresilient (PWK/PhJ, A/J, NOD/ShiLtJ, and CAST/EiJ) to Plasmodium chabaudi. Metabolites that differ significantly (*P* < 0.05 with Bonferroni correction) between resilient and nonresilient strains are shown. Download Table S2, DOCX file, 0.02 MB. Copyright © 2021 Davis et al. 2021 Davis et al. https://creativecommons.org/licenses/by/4.0/ This content is distributed under the terms of the Creative Commons Attribution 4.0 International license.

### Correlations identify metabolic markers for disease severity in P. chabaudi malaria.

**FIG 4:**
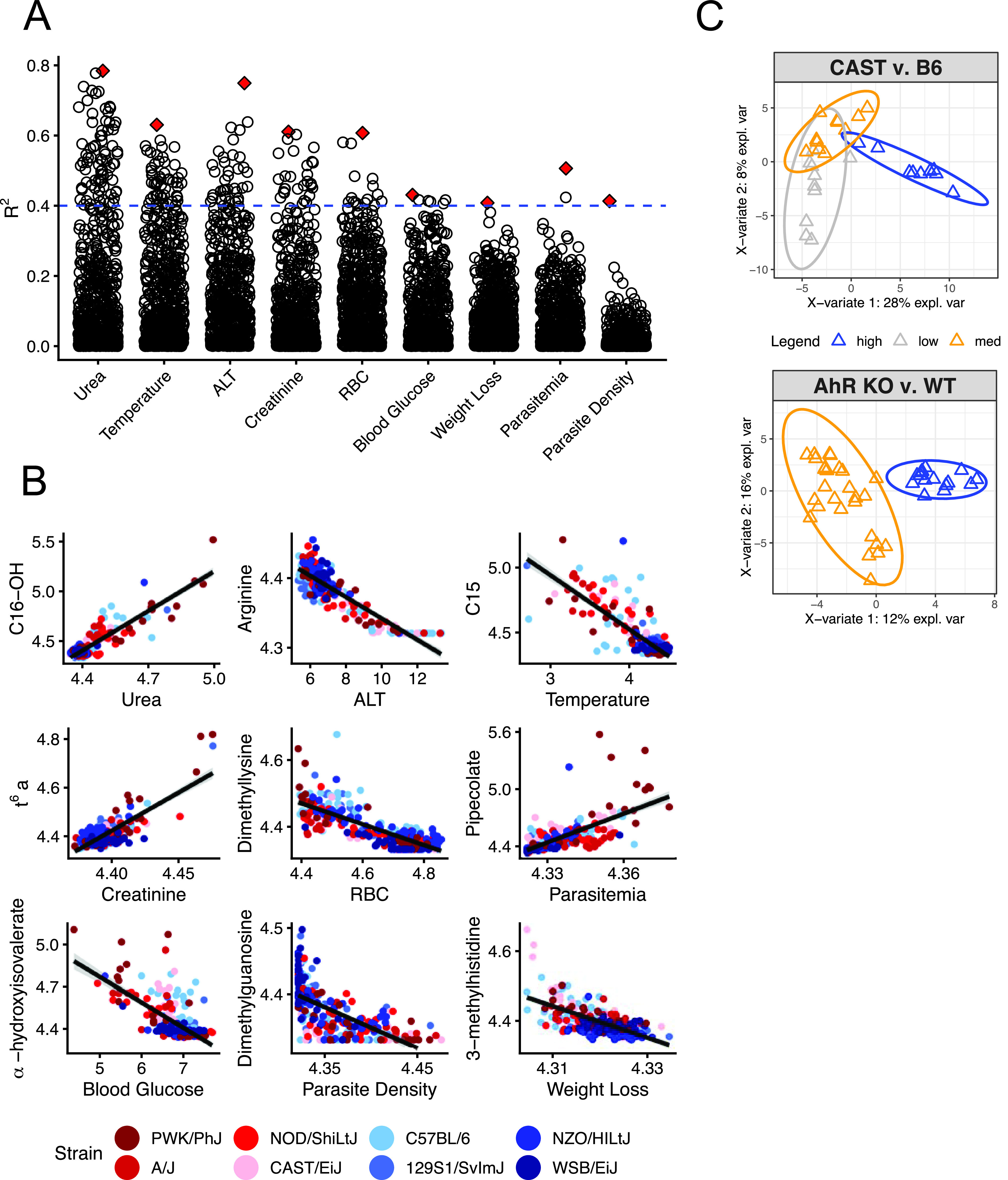
Metabolic indicators of disease severity in P. chabaudi-infected mice. (A) Log_2_-transformed scaled imputed ion counts (*n* = 751 metabolites) were correlated with the nine metrics of disease severity shown in Fig. 1. Each point represents the *R*^2^ value of the Pearson correlation for each metabolite and health combination (*n* = 751 × 9 total correlations). Red diamonds indicate the best correlation for each health metric. The dashed blue line is the threshold for *R*^2^ = 0.4. (B) Correlation plots with best-fit lines for red diamonds in panel A. C16-OH, hydroxypalmitoylcarnitine; C15, pentadecanoylcarnitine; TMAP, *N*,*N*,*N*-trimethyl-l-alanyl-l-proline betaine. From left to right, the *R*^2^ values for the graphs in the different rows are as follows: top row, 0.78, 0.63, and 0.75; middle row, 0.61, 0.61, and 0.43; bottom row, 0.41, 0.51, and 0.41. (C) sPLS-DA separates samples (triangles) from P. chabaudi-infected C57BL/6 and CAST/EiJ mice on the basis of liver damage (high [blue], moderate [orange], and low [gray]). A similar analysis separates samples from P. chabaudi-infected *Ahr^+/+^* and *Ahr^−/−^* C57BL/6 mice on the basis of liver damage (high and moderate liver damage, respectively). CAST v. B6, CAST/EiJ versus C57BL/6; AhR KO v. WT, AhR knockout versus wild type; expl. var., explained variation.

Having determined that the sickest mice displayed the greatest changes in plasma metabolome composition, we next obtained a more granular understanding of pathology-metabolism relationships by correlating individual metabolites with our nine metrics of disease (Fig. 4A). The kidney dysfunction marker urea had the highest number of strong correlations (*R*^2^ > 0.4), followed by ALT, creatinine, and temperature loss. This is consistent with results from our PCA, which identified metabolic markers for organ dysfunction as strong drivers of variation in PC1. Anemia and hypoglycemia also correlated well with some plasma metabolites, while few metabolites correlated well with weight loss, parasitemia, or parasite density. Altogether, our correlations suggest the host is a stronger driver of infection-induced plasma metabolic changes than the parasite.

Some metabolites correlated well with multiple disease metrics. For example, α-hydroxyisovalerate, a hallmark of defective leucine metabolism (33) and the best indicator of hypoglycemia in our malarial mice (Fig. 4B), correlated well with all disease metrics except parasite load (see Data Set S2 in the supplemental material). Similarly, elevated long-chain acylcarnitines were the best predictors of high plasma urea levels and temperature loss (Fig. 4B), but many also positively correlated with ALT. Long-chain acylcarnitines are also elevated in other pathological states like lipopolysaccharide (LPS)-induced inflammation (34) and acute kidney injury (35) (Fig. S2). Levels of α-hydroxyisovalerate and long-chain acylcarnitines also increase during starvation (36, 37) (Fig. S2), which is consistent with our previous observations of P. chabaudi-induced anorexia (11). Collectively, these multicorrelative metabolites may point to shared underlying mechanisms of metabolic disruption across disease states.

10.1128/mBio.02424-21.5 FIG S2 Acylcarnitines are elevated in diverse disease states. Three data sets were analyzed in addition to our own: LPS-injected female C57BL/6 mice (“LPS”, from reference 34), male Wistar rats with acute kidney injury (“AKI”, from reference 35), and female C57BL/6 mice under fasting conditions (“Starvation”, from reference 36). These were compared to P. chabaudi-infected C57BL/6 (“BL6”) and CAST/EiJ (“CAST”) mice from our study on day 9 postinfection. Log_2_ fold change in metabolite concentration (relative to healthy, uninfected or untreated mice from the source data set) was calculated for each metabolite. Gray cells indicate missing data. Download FIG S2, EPS file, 0.08 MB. Copyright © 2021 Davis et al. 2021 Davis et al. https://creativecommons.org/licenses/by/4.0/ This content is distributed under the terms of the Creative Commons Attribution 4.0 International license.

10.1128/mBio.02424-21.9 DATA SET S2 Correlations between disease severity and metabolites. This file contains the correlation information for each unique combination of disease severity metrics and metabolites shown in Fig. 3. It contains *R*, *R*^2^, and *P* values, as well as metabolite metadata. Download Data Set S2, XLSX file, 0.8 MB. Copyright © 2021 Davis et al. 2021 Davis et al. https://creativecommons.org/licenses/by/4.0/ This content is distributed under the terms of the Creative Commons Attribution 4.0 International license.

In contrast to metabolites that were broadly indicative of severe disease, some metabolites specifically correlated with just one pathology. Some of these associations have been identified previously, including pipecolate as a marker for parasitemia (38) and *N*,*N*,*N*-trimethyl-alanylproline betaine, which positively correlates with creatinine in our data and was recently identified as a sensitive marker for kidney injury (39). Other metabolites suggested directions for future study. For example, 3-methylhistidine, a proposed marker for muscle protein breakdown (40), correlated with weight loss in our malarial mice. Muscle protein breakdown has not been reported in P. chabaudi-infected mice, but cachexia occurs in other inflammatory conditions (18, 41, 42). We also noted a strong association between arginine depletion and elevated ALT (Fig. 4B), a molecular proxy for liver damage (6, 7). While both abnormalities are prevalent in human malaria and pose health concerns (7, 20, 43–46), the connection between liver damage and arginine in malaria is understudied.

### Arginine depletion is a specific indicator of liver damage in P. chabaudi malaria.

To better determine how specifically plasma arginine depletion indicates liver damage, we applied sparse partial least-squares discriminant analysis (sPLS-DA) to identify the best metabolic markers for high ALT in our data and in a second data set (19). sPLS-DA is a supervised learning method that selects variables that best differentiate samples based on user-assigned groups and is well-suited to sparse and heterogeneous multi-omics data (47–49) (Materials and Methods). We limited analysis in our data set to two strains of mice—CAST/EiJ and C57BL/6—that had low and high levels of ALT, respectively (*n* = 5 mice per strain per day on days 7 to 9) but similar levels of other disease severity metrics. By comparing samples from only these two strains, we reasoned we could identify metabolites that are specific to liver damage rather than other pathologies. Our sPLS-DA model identified arginine, adenosine, and methionine along with other metabolites in component 1 as best able to discriminate among samples with “high,” “medium,” or “low” ALT values (Fig. 4C, top, and Table S3). We performed a similar analysis (Fig. 4C, bottom) on plasma metabolomes from P. chabaudi-infected wild-type and *Ahr^−/−^* C57BL/6 mice (*n* = 6 mice per genotype per day for days 6 to 8 postinfection), which have high and low levels of ALT during infection, respectively. Upon comparing metabolic candidates from sPLS-DA and our correlation analysis (*n* = 40 to 50 metabolites for each of three analyses), we found that overlap between the three analyses was low—just one to three metabolites were identified by any two of three analyses—but arginine was selected by all three (Fig. 4C, Data Set S2, and Table S3).

10.1128/mBio.02424-21.3 TABLE S3 Metabolites identified by sPLS-DA. Metabolites that stably separate CAST/EiJ from C57BL/6 samples or *Ahr^+/+^* C57BL/6 from *Ahr^−/−^* C57BL/6 samples on the basis of liver damage using sPLS-DA. “Freq” refers to the frequency with which subsamples of data select a given metabolite during leave-one-out cross-validation (1.00 = 100% of the time). Only metabolites with a frequency (Freq) of ≥0.9 are shown. Download Table S3, DOCX file, 0.03 MB. Copyright © 2021 Davis et al. 2021 Davis et al. https://creativecommons.org/licenses/by/4.0/ This content is distributed under the terms of the Creative Commons Attribution 4.0 International license.

### Malarial liver damage releases hepatic Arg1 into circulation in conjunction with arginine depletion.

**FIG 5:**
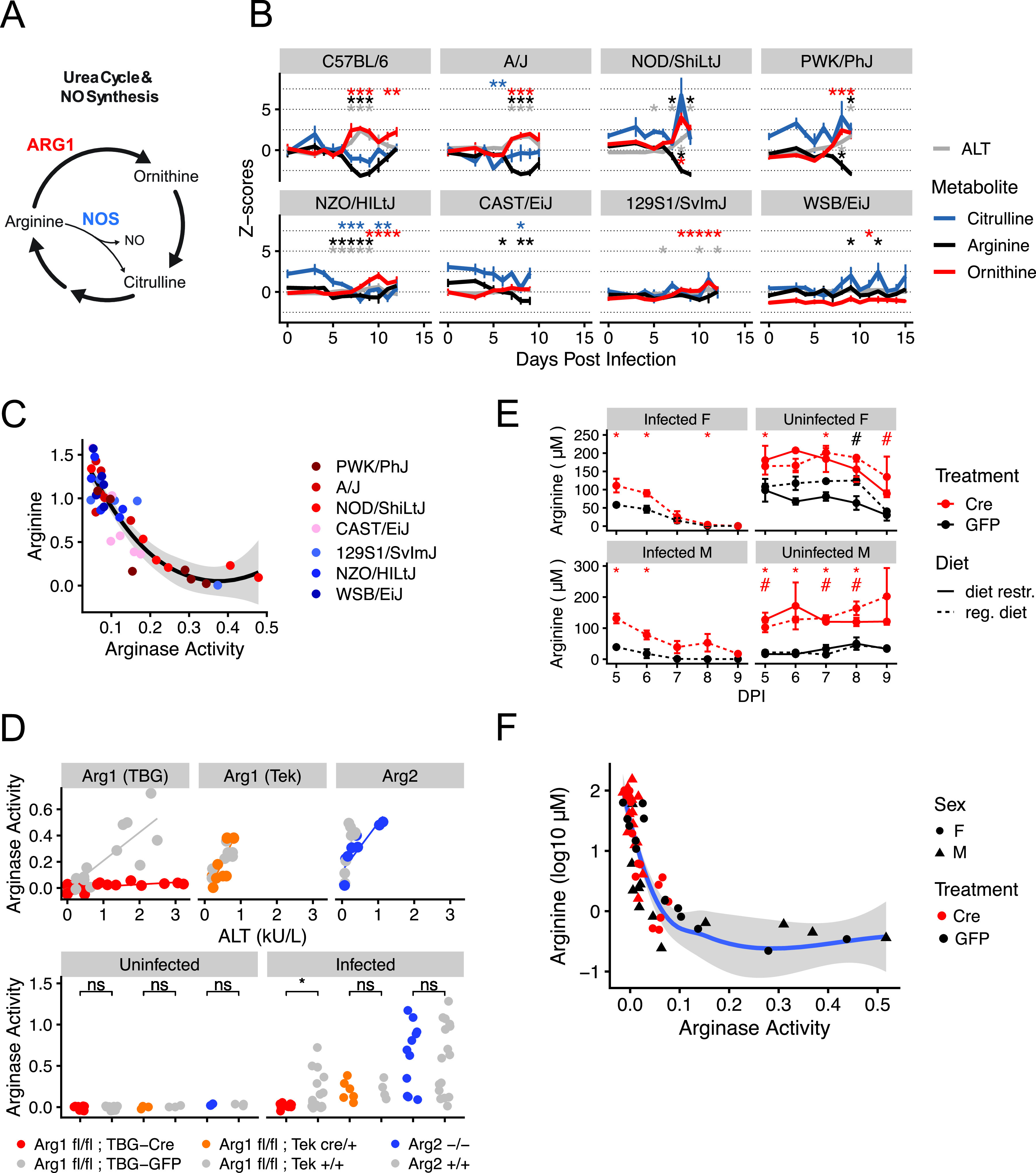
The liver controls arginine metabolism via arginase-1. (A) A schematic of the urea cycle, polyamine metabolism, and nitric oxide production. NO, nitric oxide; NOS, nitric oxide synthase; ARG1, arginase-1. (B) Median scaled imputed ion counts of arginine, ornithine, and citrulline throughout infection. Z-score = 1 standard deviation from the mean of uninfected C57BL/6 mice. *n* = 5 animals per strain per day. (C) Plasma arginase activity (nanomoles of H_2_O_2_/minute/microliter of blood) and scaled imputed ion counts of arginine (log_10_-transformed Z-scores) in seven of eight P. chabaudi-infected founder strains (*n* = 6 per strain). A Loess curve with standard errors (gray shaded area) is shown. (D) Plasma arginase activity (nanomoles of H_2_O_2_/minute/microliter of blood) and ALT in arginase knockout C57BL/6 mice before and during P. chabaudi infection (*n* ≥ 3 uninfected mice per genotype, *n* ≥ 6 infected mice per genotype in two experimental replicates). Statistical significance by Welch’s *t* test is indicated as follows: *, *P* < 0.05; ns, not significant. (E) Mean (± standard error [SE]) plasma arginine in uninfected (with or without dietary restriction) and P. chabaudi-infected *Arg1^fl/fl^; TBG-Cre* and *Arg1^fl/fl^; TBG-GFP* mice (*n* = 3 per condition per sex, day 5 postinfection is day 12 post-AAV injection). F, female; M, male; diet restr., dietary restriction; reg. diet, regular diet. (F) Plasma arginase activity (nanomoles of H_2_O_2_/minute/microliter of blood) and arginine for infected mice and days shown in panel E. A Loess curve with standard errors (gray shaded area) is shown.

In healthy, ureotelic animals, hepatic arginase-1 (Arg1) converts arginine to ornithine and urea (50) (Fig. 5A) in a process that is spatially restricted to hepatocytes. However, drug-induced hepatocellular injury releases hepatic arginase-1 into circulation where it depletes plasma arginine (6). Only associative evidence has linked arginine depletion with hepatic arginase and liver injury in malaria (44). Given the strong and specific association between arginine depletion and ALT in our data, we hypothesized that P. chabaudi-induced liver damage depletes plasma arginine by releasing hepatic Arg1 into circulation. In support of our hypothesis, we found that the arginase product ornithine is elevated in mice with high ALT and low arginine (Fig. 5B, top row, C57BL/6, A/J, NOD/ShiLtJ, and PWK/PhJ, *n* = 3 to 5 mice per strain per day). In contrast, the urea cycle metabolite and nitric oxide synthase product citrulline varied independently of arginine depletion and ALT. Further analysis of founder strain samples revealed a positive correlation between plasma arginase activity and plasma ALT (*R*^2^ = 0.75 [Fig. 5C]).

Plasma arginase activity is increased in malaria patients, but it has been attributed to other sources of Arg1 such as red blood cells or monocytes (44, 45). The other mammalian arginase arginase-2 (Arg2), which is highly expressed in immune cells as well as the gut and kidney (51–53), is rarely considered (45). To determine the isoform and tissue source of plasma arginase during P. chabaudi infection, we utilized a globally Arg2-deficient and two tissue-specific Arg1-deficient C57BL/6J mouse strains. Because murine Arg1 deletion results in lethality 14 days after birth (54), we crossed Arg1 floxed mice (*Arg1^fl/fl^* mice [16]), with *Tek-Cre* mice, generating *Arg1^fl/fl^; Tek-Cre* mice that lack Arg1 specifically in blood cells and the endothelium. To overcome lethality caused by liver-specific Arg1 deficiency (55), we injected *Arg1^fl/fl^* mice with adeno-associated viral (AAV) vectors that express Cre (or green fluorescent protein [GFP] for control animals) under the control of the liver-specific *TBG* promoter. This yielded *Arg1^fl/fl^; TBG-Cre* mice that display hyperargininemia and reduced plasma arginase activity at 2 weeks postinjection and that die from liver Arg1 deficiency at 3 weeks postinjection (Fig. S3A and S3B).

10.1128/mBio.02424-21.6 FIG S3 Arginase, arginine, and diet dynamics in arginase knockout mice. (A and B) Mice were injected with increasing doses of TBG-Cre (*Arg1^fl/fl^; TBG-Cre* mice) or a high dose of TBG-GFP (*Arg1^fl/fl^; TBG-GFP* mice) (*n* = 2 to 3 per treatment). (A) Levels of arginine after AAV injection (day −7) in uninfected mice. Day 0 corresponds to infection day for infected mice in panel B. (B) Arginase activity on day 8 post-P. chabaudi infection (*n* = 3 per group. One mouse in the high- and medium-dose treatments died prior to day 8.) (C) Plasma arginase activity (nanomoles of H_2_O_2_/minute/microliter of blood) and arginine for infected mice on days7 to 9 postinfection (one representative experiment for *Arg1^fl/fl^; Tek-Cre* and *Arg1^fl/fl^* mice and two experimental replicates for *Arg2^+/+^* and *Arg2^−/−^* mice. (D and E) Male and female mice (*n* = 3 uninfected and *n* = 4 infected) were injected with high-dose TBG-Cre (red) or TBG-GFP (black). (D and E) Food intake (D) and body weight (E) were measured. Each uninfected mouse with dietary restriction (diet restr.) was matched by age, sex, and genotype to a P. chabaudi-infected mouse and was allowed to eat only as much as its corresponding infected mouse. Infected mice and a subset of uninfected mice were fed *ad libitum* (regular diet [reg. diet]). Download FIG S3, EPS file, 0.1 MB. Copyright © 2021 Davis et al. 2021 Davis et al. https://creativecommons.org/licenses/by/4.0/ This content is distributed under the terms of the Creative Commons Attribution 4.0 International license.

We infected these knockout mice and their respective controls (*n* = 3 mice per genotype per experiment with two experimental replicates) with P. chabaudi and measured ALT and plasma arginase activity at peak infection intensity. As expected, uninfected mice of all genotypes exhibited low ALT and arginase activity. In infected, nonknockout mice, ALT and arginase activity positively correlated (Fig. 5D). ALT and arginase activity also correlated in *Arg2^−/−^* and *Arg1^fl/fl^; Tek-Cre* mice, suggesting neither Arg1 in the blood and endothelium nor Arg2 contribute to plasma arginase activity during P. chabaudi infection. In contrast, *Arg1^fl/fl^; TBG-Cre* mice exhibited high levels of ALT but significantly reduced plasma arginase activity relative to infected *Arg1^fl/fl^; TBG-GFP* mice (Fig. 5). This suggests hepatic Arg1 is the source of plasma arginase activity in P. chabaudi-infected mice.

Low plasma arginine is associated with increased vascular stress (44) and negative pregnancy outcomes in malaria patients (46). However, arginine infusion in malaria patients sometimes fails to restore arginine levels and mitigate vascular stress (56), motivating further exploration of the causes of hypoargininemia. Plasma arginase is associated with arginine depletion in malaria (44, 45), which is consistent with our hypothesis that hepatic Arg1 depletes arginine. Infection-induced anorexia and altered flux of arginine through the urea cycle are other explanations that accounted for some but not all arginine depletion in mice with experimental cerebral malaria (57). To assess the importance of these factors on plasma arginine dynamics, we restricted food intake in uninfected *Arg1^fl/fl^; TBG-Cre* and *Arg1^fl/fl^; TBG-GFP* mice to the level of age- and sex-matched P. chabaudi-infected mice (Fig. S3C, *n* = 4 infected and 3 uninfected mice per sex, sampled longitudinally). Despite comparable weight loss in infected and uninfected mice (Fig. S3C), decreases in their plasma arginine during dietary restriction were relatively modest; only infected mice displayed dramatic hypoargininemia (Fig. 5E). Arginine depletion correlated strongly with plasma arginase activity in both genotypes, even in *Arg1^fl/fl^; TBG-Cre* mice with significantly reduced arginase activity (Fig. 5F), suggesting that even residual amounts of circulating arginase are sufficient to reduce plasma arginine by 100-fold or more. Finally, given the dramatic elevation of plasma arginine following knockdown of hepatic Arg1 (Fig. S3A and Fig. 5E) (58), it is unlikely that this type of urea cycle disruption contributes to arginine depletion. Collectively, our data suggest that hepatic Arg1 maintains plasma arginine homeostasis and that circulating hepatic Arg1 depletes plasma arginine during P. chabaudi infection.

### Metabolic changes in murine malaria recapitulate facets of human malaria.

Serum metabolites change dramatically in human malaria patients, which led us to ask if P. chabaudi infection shares metabolic features with P. falciparum infection. However, prior to a direct comparison between human and mouse metabolic changes, we must acknowledge differences between human malaria and our murine model. First, human and rodent infections are caused by different parasites, P. falciparum and P. chabaudi, respectively. P. falciparum can cause cerebral malaria and death in humans (2), while lethal cerebral malaria in mice is restricted primarily to Plasmodium berghei infection models; in contrast, P. chabaudi-induced death is thought to be due to severe anemia. Furthermore, P. falciparum infections are self-resolving while mice continue to carry P. chabaudi, albeit at dramatically reduced levels. Of particular relevance for vascular health, P. falciparum protein PfEMP1 mediates cytoadherence of parasites to the vascular endothelium (3), which is associated with inflammation and vascular stress. Less is known about cytoadherence in P. chabaudi, though it is thought to occur (59). Finally, natural *Plasmodium* infection begins when parasite sporozoites infect the host liver, while our murine model bypasses the sporozoite stage. Nonetheless, the sporozoite stage of infection is largely symptomatically silent. Liver damage in human malaria occurs later in infection, during the acute blood stage and concurrently with parasite clearance (7). This is consistent with our findings in P. chabaudi-infected mice (Fig. 1). Similarly, most metabolic studies of humans, including the data presented below, sample the blood stage of infection when severe-disease-like liver dysfunction and acute kidney injury are most evident.

**FIG 6:**
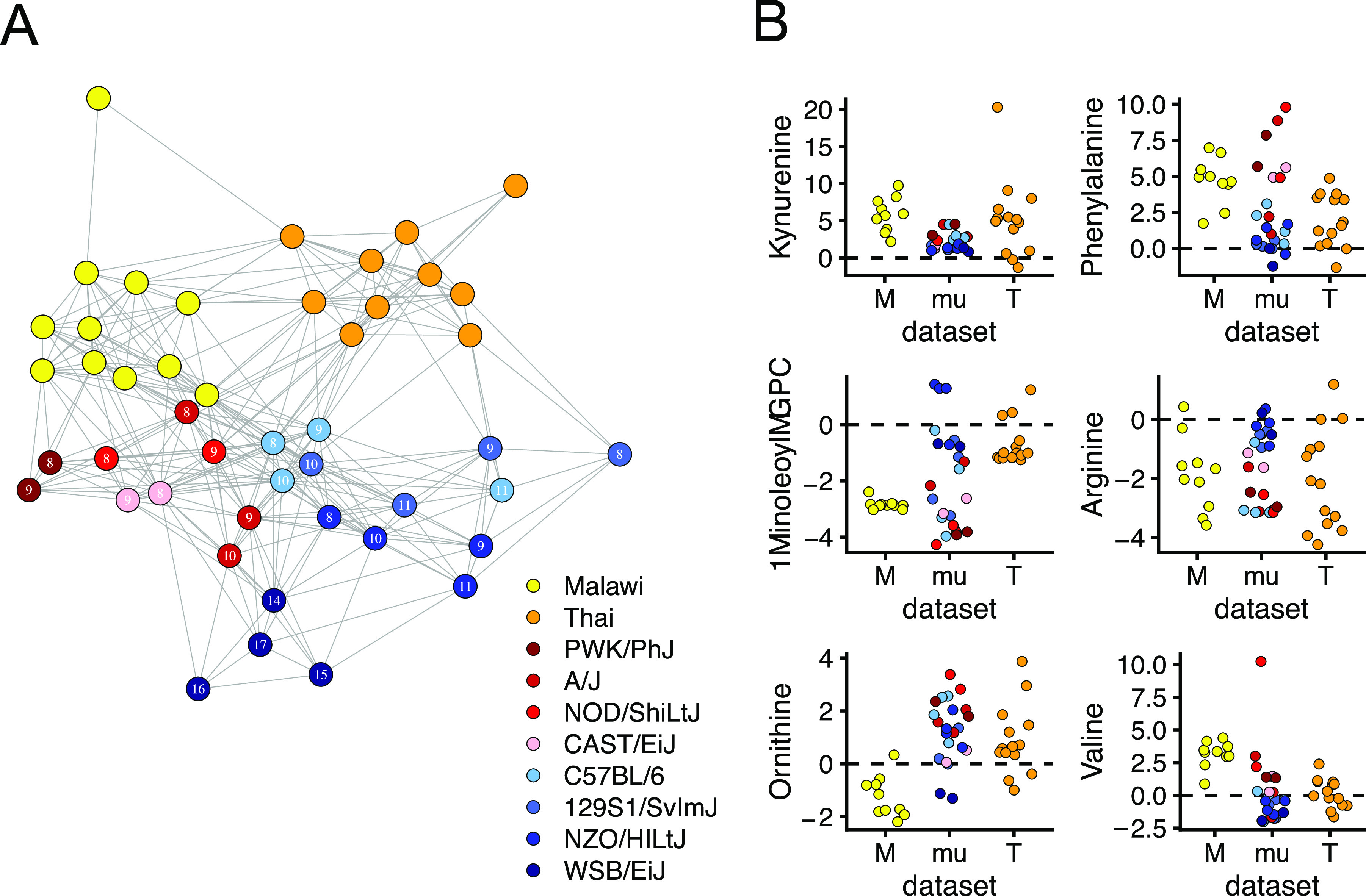
Mice and humans share plasma metabolic features of *Plasmodium* infection. A mouse-human correlation network highlights similarity of mouse and human metabolic responses to malaria. Two human metabolic data sets from Malawian cerebral pediatric P. falciparum-infected malaria patients (yellow) (*n* = 10) and adult Thai severe P. falciparum-infected malaria patients (orange) (*n* = 10) were analyzed. (A) The 42 metabolites (values Z-scored from uninfected mice or patients) measured in all three data sets were included in a sample-sample interdata set correlation analysis. The resulting network is shown. Each sample is a network node, and edges were drawn between any sample with *R*^2^ ≥ 0.5. Mouse samples are the median values for each strain (days 8 to 11 or 14 to 17 for WSB/EiJ) postinfection. Each symbol represents the value for a sample from one individual. (B) The same data are used to display for six individual metabolites. M, Malawian; mu, murine; T, Thai.

To metabolically compare murine and human malaria, we started with publicly available metabolic data from two populations of P. falciparum-infected individuals: Thai adults with uncomplicated malaria (60) (*n* = 10) and Malawian pediatric patients with severe cerebral malaria (20) (*n* = 10). Using metabolites that were measured in both human studies and our mouse study (*n* = 42, predominantly amino acids and lipids), we created a network using the correlation matrix of mouse samples at peak disease severity (using median metabolite values from five mice per day) and all human samples (Fig. 6A). In the network, mice with mild disease were better connected to Thai adults with uncomplicated malaria, while mice with severe disease correlated better with Malawian pediatric patients with severe disease. A subset of mouse samples (C57BL/6 at peak infection severity) was highly connected to samples from both human data sets, suggesting they shared similarity with both human populations.

To understand which metabolic changes drove interspecies similarity in the correlation network, we compared the levels of individual metabolites across our mice and the human samples (Fig. 6B and Fig. S4). In most mice and humans, infection significantly increased levels of phenylalanine and kynurenine and decreased levels of arginine, glycine, serine, and long-chain glycerophosphatidylcholines (GPCs). Notably, some metabolites like valine and 1-linoleoyl-GPC changed more in severe disease (Malawian patients and less resilient mouse strains [red]) than in mild disease (Thai patients and resilient mouse strains [blue]). For many other metabolites, the two human data sets qualitatively differed, while mice tended to either match one population or display intermediate responses (Fig. 6B). For example, ornithine was elevated in Thai patients and our mice but depleted in Malawian patients. Conversely, valine was elevated in Malawian patients and mice but relatively unchanged in Thai patients.

10.1128/mBio.02424-21.7 FIG S4 Mice and humans share plasma metabolic features of *Plasmodium* infection. Individual values for each metabolite shown in Fig. 6. for all three data sets. Mouse samples in the network are derived from the samples highlighted here in gray boxes. Human Malawian samples are paired samples from patients during two hospital visits, an initial visit during acute infection (initial [Init]) and a follow-up (FU) visit during convalescence. Human Thai samples are P. falciparum-infected individuals (Pf) and healthy controls (HC). Welch’s *t* test (paired for Malawian patients, uninfected versus infected for Thai patients and mouse samples): *, *P* < 0.05. Download FIG S4, EPS file, 0.7 MB. Copyright © 2021 Davis et al. 2021 Davis et al. https://creativecommons.org/licenses/by/4.0/ This content is distributed under the terms of the Creative Commons Attribution 4.0 International license.

## DISCUSSION

Host metabolism changes in response to infection, but we still know little about how host metabolism responds to severe versus mild disease. To better understand how disease severity affects host metabolism in murine malaria, we monitored disease severity metrics (Fig. 1) and plasma metabolites in eight genetically diverse mouse strains that vary widely in survival following acute Plasmodium chabaudi infection. While P. chabaudi primarily causes nonfatal severe anemia due to high parasitemia, we observed a wide range of other disease severity phenotypes in which parasitemia, anemia, temperature loss, weight loss, liver and kidney injury, and hypoglycemia were generally more severe in mice that succumb to infection. We also observed a wide range of metabolic responses and used PCA to show that the magnitude of metabolic response scaled with overall disease severity (Fig. 3).

Previous human studies (e.g., references 4, 5, and 21) also reported malaria severity-dependent changes in host metabolism. To our knowledge, Leopold et al. provide the most comprehensive picture of these changes in Bangladeshi patients, both in terms of the number of metabolites assessed and in number of facets of disease severity examined, including acute kidney injury, metabolic acidosis, and coma (4, 5). Our work complements these studies by including additional metrics of disease severity with quantitative data for the degree of disease severity. Using our approach, we showed that malarial mice and humans share some plasma metabolic changes during infection (Fig. 6). These conserved metabolic changes fell into one of two broad categories: responses consistent in all three populations, and those shared by mice and just one of the two human populations.

All hosts shared some malaria-induced metabolic changes. Kynurenine and phenylalanine were elevated in all individuals, mouse and human. Kynurenine is a tryptophan breakdown product and ligand of the aryl hydrocarbon receptor (AhR), which protects the host in multiple models of malaria and sepsis (19, 61, 62). Phenylalanine is also elevated during infection, as in other malaria studies (4, 63, 64). Phenylalanine elevation may be caused by increased protein catabolism (57) and malaria-induced depletion of tetrahydrobiopterin (63), a cofactor required for enzymatic conversion of phenylalanine to tyrosine (65). Arginine was depleted in most individuals, which is consistent with all mouse and human studies we examined (4, 5, 20, 21, 43, 44, 57, 66). Finally, most infected mice and humans had depleted long-chain GPCs (also observed in references 21 and 22), which may reflect changes in cell membranes, cell membrane lysis, or altered lipid metabolism. The nonessential amino acids glycine and serine, which P. falciparum uses in folate derivative-dependent DNA synthesis (67), were also depleted. Given the similarities we identified between humans and mice, our work supports the use of P. chabaudi-induced murine malaria as a model to understand host metabolic responses to malaria.

For a number of metabolites, mice and one human population differed from the second human population. For instance, ornithine was generally higher in our malarial mice and in Thai adults with uncomplicated malaria (60) but low in Malawian children with cerebral malaria (20). We anticipate elevated plasma arginase explains ornithine elevation in malarial humans as it does in our murine data. Like ornithine, long-chain acylcarnitines (ACs) (e.g., C16 and C18) were also elevated in malarial mice and Thai patients but depleted in Malawian patients (data not shown). We found that long-chain ACs correlated with many pathologies in our model, including liver and kidney dysfunction and temperature loss. Others have also noted elevation of long-chain ACs following acute kidney injury, fasting, and LPS injection in mice (see Fig. S2 in the supplemental material) (34–36). Fasting elevates long-chain ACs and is usually accompanied by an increase in fatty acid β-oxidation gene expression (36). However, in infection (34) and long-chain fatty acid oxidation disorders (68), β-oxidation is suppressed. Measuring fatty acid β-oxidation gene expression in P. chabaudi-infected mice would help determine whether elevated long-chain ACs in our model indicate a normal fasting response or impaired β-oxidation. We also found that the branched-chain amino acids (BCAAs) leucine, isoleucine, and valine were elevated in mice with severe disease. Leopold et al. (4) also noted BCAA elevation in patients with severe but not uncomplicated malaria, which is consistent with our mice and the two human data sets examined here. BCAAs are altered in fasting states in both mice and humans (36). Though not present in either human data set, α-hydroxyisovalerate was a sensitive marker for hypoglycemia and weight loss in our murine data, and it is also indicative of defective leucine metabolism (33). Collectively, these data suggest infection-induced anorexia or changes in energy metabolism produce a strong molecular signature in severe malaria.

Our data suggest that diverse mice capture more of the diversity of human malaria responses than one human population alone can. However, the small number of metabolites (42 metabolites), patients (*n* = 10 for each of two human data sets), and data sets (3 data sets) included in this study limit our ability to make broad generalizations. There is significant variability in metabolic responses in humans (Fig. S4), which may be due to any number of confounding factors, including differences in age, geography, malarial syndrome, disease severity, study design, and instrumentation. We selected these data sets because they had the highest number of metabolites available for comparison with our murine data, but we recognize that we cannot definitively establish causal links between disease severity and metabolic responses in these data sets. To increase robustness in linking metabolic change with disease severity in humans, future studies would benefit from measuring samples from multiple mouse and human populations in tandem, alongside careful measurements of disease severity in both species like we present in Fig. 1 and like those presented in the works of Leopold et al. (4, 5).

In addition to metabolites that generally indicate severe disease, several molecules from our study associated with individual pathologies (Fig. 4). Two of these correlations corroborated known associations: pipecolate as a biomarker for parasitemia (38), and *N*,*N*,*N*-trimethyl-alanylproline betaine (TMAP), which correlated with the kidney injury marker creatinine and was recently identified as a sensitive marker of kidney dysfunction (39). Other correlations like the association of 3-methylhistidine with weight loss and the correlation between α-hydroxyisovalerate and hypoglycemia are consistent with previous reports of muscle protein catabolism elevating plasma 3-methylhistidine (40) and of defective leucine metabolism causing hypoglycemia and α-hydroxyisovalerate elevation (33). Together, these findings support the validity of our correlation method and motivate further study of associations like dimethyllysine and red blood cells (Fig. 4B), *N*^6^-threonylcarbamoyladenosine (t^6^A) and creatinine (Fig. 4B), acylcarnitines (Fig. 4B) as discussed above, and others.

We demonstrated that our bioinformatic approach yielded precise and testable mechanistic hypotheses. Specifically, our correlation analysis pointed to the liver, rather than red blood cells, immune cells, or the parasite as the source of arginine-depleting arginase (Fig. 4B and C). We tested this hypothesis by measuring plasma arginase and arginine in liver-specific Arg1 knockout mice, blood- and endothelium-specific Arg1 knockout mice, and Arg2 knockout mice on a C57BL/6 background. In support of our bioinformatic data, we found that plasma arginase activity in malaria was reduced only in liver-specific Arg1 knockout mice. These results challenge previous hypotheses about red blood cell, monocyte, or endothelial sources of Arg1 in malaria. *Plasmodium* parasites also carry genes that encode an arginase that significantly depletes arginine *in vitro* (69) but not plasma arginine *in vivo* (57). Consistent with these studies, our results suggest that the host, rather than the parasite, is the source of plasma arginase and arginine depletion in malaria. This, along with the relatively few strong parasite-metabolite correlations we observed, suggests the host is more directly responsible than the parasite for driving plasma metabolic changes in acute malaria.

Many studies observe decreased plasma arginine during infection (2, 4, 5, 22, 43–46, 56, 57, 70, 71) and even attempt to restore levels of this vasoprotective amino acid (43, 56, 66), but the cause(s) of arginine depletion in malaria is still debated. Anorexia and urea cycle changes accounted for some but not all infection-induced plasma arginine depletion in mice with experimental cerebral malaria (57). Our data support a minor role for anorexia in depleting plasma arginine and suggest that disrupting the urea cycle by hepatic Arg1 knockout does not deplete arginine but rather increases it. Instead, our data suggest that even small amounts of circulating hepatic Arg1 can dramatically deplete plasma arginine in diverse mice. Thus, arginase may be relevant even at the low concentrations seen in the P. berghei model (72, 73).

We did not identify any single factor that is capable of maintaining normal arginine levels in malarial mice; even *Arg1^fl/fl^; TBG-Cre* mice (with significantly reduced plasma arginase activity) eventually suffered from hypoargininemia. Thus, despite our strong correlative evidence for small amounts of circulating hepatic Arg1 depleting arginine, this raises the possibility that there remains an unidentified factor that is the direct cause of arginine depletion. To better delineate the arginine-depleting role of hepatic Arg1 relative to other factors, future studies may benefit from careful kinetic analysis of arginine depletion following arginine infusion in P. chabaudi-infected *Arg1^fl/fl^; TBG-Cre* and *Arg1^fl/fl^; TBG-GFP* mice, which theoretically differ only in their levels of circulating plasma arginase.

Like arginine depletion (Fig. 6), liver damage is prevalent but not universal in malaria patients (7). Failure to account for differences in liver damage and plasma arginase activity may explain why arginine infusion does not always restore plasma arginine in malaria patients (43, 56). While liver damage and plasma arginase activity did not predict survival across the eight mouse strains used in our study, increased plasma arginase and arginine depletion are associated with pathologies we did not measure, including vascular stress (44, 74), negative birth outcomes in pregnant women (46), and gut vascular permeability in *Plasmodium*-Salmonella coinfection (71). Collectively, our data motivate further study of the relationships between liver damage, arginase, and vascular health in infectious disease. C57BL/6 and CAST/EiJ mice, which had high and low ALT, respectively, may provide a useful model for those studies.

### Future directions.

Should liver damage emerge as an important player in hypoargininemia in human malaria, NETosis (75) and IL-1-mediated inflammation (76) may be therapeutic targets for limiting liver damage. Given the dramatic differences in plasma immune cell and cytokine responses between C57BL/6 and CAST/EiJ mice (Fig. 2), these two strains may provide a natural study system for further dissection of immunopathology in the liver. Future efforts might also determine why some nonresilient mouse strains had weak overall cytokine responses, while others had hyperinflammatory responses. These data point to a trade-off between effective immunity and immunopathology in malaria that should be explored. We characterized other strain-specific responses that suggest non-C57BL/6 mouse strains will be the best model for some malarial host responses. The P. chabaudi model does not recapitulate some aspects of P. falciparum infection, perhaps most notably cerebral symptoms. Future metabolic studies would benefit from using the P. berghei murine model of cerebral malaria. We used genetically diverse mice because they displayed high phenotypic diversity, but we did not determine how genetics contributed to phenotypic diversity. Quantitative trait locus mapping in malarial DO mice would achieve that goal. Finally, the insights we gained in this study relied heavily on correlative data that do not link processes across time. To identify metabolic processes that connect early, acute, and late parts of the host response (19), longitudinal metabolic sampling of diverse mice would enable use of predictive tools like hidden Markov models.

### Implications and conclusion.

We saw that humans and diverse mice exhibit variation in metabolic responses to malaria that depends on disease severity. This implies that future metabolic studies of infection should account for both the nature and severity of disease. We used diverse mice to highlight the variability of metabolic responses and disease severity following infection and used this variability to yield new insights about liver damage and arginine metabolism. P. chabaudi is not a perfect model of human malaria, but it recapitulates some important facets of human disease. We view our work with diverse mice as a first step toward determining which human malarial metabolic changes can be studied in the P. chabaudi model. Collectively, our data provide new bioinformatic and mechanistic insights into infection metabolism and motivate the use of genetically and phenotypically diverse mice in future studies.

## MATERIALS AND METHODS

### Ethics statement.

Mouse studies were conducted at Stanford University in accordance with NIH guidelines, the Animal Welfare Act, and federal law. They were approved by the Animal Care and Use Committee. Stanford University is accredited by the International Association for Assessment and Accreditation of Laboratory Animal Care (AALAC). All animal work was done according to protocols approved by Stanford University’s Administrative Panel on Laboratory Animal Care (APLAC) and overseen by the Institutional Animal Care and Use Committee (IACUC) under protocol identifier (ID) 30923 (D. S. Schneider).

### Parasites.

Plasmodium chabaudi *chabaudi* AJ (Malaria Research and Reference Resource Center [MR4]) was tested for contaminating pathogens prior to use.

### Mice.

Wild-type mice were purchased from the Jackson Laboratory (Bar Harbor, ME, USA) (WSB/EiJ stock no. 001145, 129S1/SvImJ stock no. 002448, NZO/HILtJ stock no. 002105, CAST/EiJ stock no. 000928, A/J stock no. 000646, NOD/ShiLtJ stock no. 001976, and PWK/PhJ stock no. 003715), and Diversity Outbred [stock no. 009376] [24f] Charles River [C57BL/6]). A subset of expensive mice (WSB/EiJ, NZO/HILtJ, and PWK/PhJ) were also bred in-house. Animals were maintained specific pathogen free (SPF) and housed in the Stanford Research Animal Facility according to Stanford University guidelines, accredited by the Association of Assessment and Accreditation of Laboratory Animal Care (AAALAC) International. All mouse experiments were approved by the Stanford Administrative Panel on Laboratory Care (APLAC).

AhR knockout mice were purchased from Taconic (C57BL/6-*Ahr^tm1.2Arte^*; stock no. 9166, B6-F for wild-type controls). Arg2 (*Arg2^tm1Weo^*/J, stock no. 020286) (51) knockout mice were purchased from JAX. All controls were age matched and purchased from JAX (JAX B6 stock no. 000664). Arg1 floxed mice were purchased from JAX (C.B6-*Arg1^tm1Pmu^*/J, stock no. 008817 [16]) and Cre mice [*Tie2:* B6.Cg-Tg(Tek-cre)12Flv/J, stock no. 004128] (77) were bred in-house. Arg2 mice were further bred in-house to yield littermate control animals.

### P. chabaudi infection.

Two female C57BL/6 mice were given intraperitoneal (i.p.) injections of 100 μl frozen stock of P. chabaudi-infected red blood cells (iRBCs). When parasitemia reached 10 to 20% at 8 to 10 days postinfection, mice were euthanized, and blood was obtained via cardiac puncture. Blood was diluted to 10^5^ iRBCs/100 μl in Kreb’s saline with glucose (KSG) and administered i.p. to experimental animals at a dose of 10^5^ iRBCs (78). Control animals received 100 μl of vehicle i.p. Only female mice 8 to 12 weeks of age were used for P. chabaudi experiments. Experiments were performed in multiple cohorts. Parasitemia was quantified via thin blood smear, methanol fixation, KaryoMAX Giemsa (Gibco) staining, and manual microscope counting at ×100 magnification. RBCs were quantified using a BD Accuri C6 Plus cytometer (see “Longitudinal P. chabaudi monitoring” below).

### Longitudinal P. chabaudi monitoring.

Longitudinal monitoring was performed as described previously (31). For each mouse, baseline RBC, weight, body temperature, and blood glucose measurements were collected between 1 and 5 days prior to infection. In some cases, blood glucose was collected only at baseline sampling and on the day of sacrifice. Mice were restrained during sample collection using tail-access rodent restrainers (Stoelting Co.). Blood was collected from the tail vein by nicking the end of the tail with disinfected surgical scissors, and depositing the blood into EDTA-coated capillary tubes to prevent clotting. For total RBC quantitation, 2 μl of blood was diluted in 1 ml of cold 1× Hanks’ balanced salt solution (HBSS) and kept on ice until absolute RBC counts were obtained using forward and side scatter gates on a BD Accuri C6 Plus flow cytometer. To record body temperature, mice in the metabolic screen experiments were implanted with subcutaneous electronic temperature and ID transponders (IPTT-300 transponders, Bio Medic Data System, Inc.) 1 week prior to infection. Mice were locally anesthetized using a 2% lidocaine solution (100 μg delivered per dose) prior to implantation. Temperature data were recorded using a DAS-7006/7 s reader (Bio Medic Data System, Inc.). Subsequent to metabolic screen experiments, body temperatures were measured using a thermocouple thermometer and mouse rectal probe (World Precision Instruments, RET-3). Blood glucose measurements were obtained with 2 μl of tail vein blood analyzed with a Bayer Contour Blood Glucose Monitor and Test Strips. Postinfection sampling began on day 4 or 5 postinfection. Parasitemia values were obtained as detailed above. Parasite density is the number of iRBCs per microliter of blood and is calculated by multiplying parasitemia by the number of total RBCs.

### Flow cytometry.

White blood cells, platelets, and reticulocytes were quantified in a subset of mice at baseline sampling and on odd-numbered sampling days from day 3 through day 11. Cells were first quantitated on an Accuri cytometer as detailed in “Longitudinal P. chabaudi monitoring.” RBC counts were used as an approximation for total blood cell counts. Approximately 10 million cells were plated in fluorescence-activated cell sorting (FACS) buffer (phosphate-buffered saline [PBS], 0.2% fetal bovine serum [Sigma], 5 mM EDTA). Prior to staining, the cells were incubated in TruStain FcX antibody (Biolegend) for at least 5 min at 4°C. A cocktail containing the Live/Dead Fixable Blue stain (Fisher catalog no. L34962) and antibodies against the following antigens was added to the blocked cells: CD71 peridinin chlorophyll protein (PerCP)-Cy5.5 (clone RI7217), TER-119 phycoerythrin (PE)-Cy7 (TER-119), TCRgd PE (UC7-13D5), CD19 brilliant violet 785 (BV785) (6D5), CD3 BV650 (17A2), CD8 BV510 (53–6.7), Ly6G BV421 (1A8), CD4 Alexa Fluor 700 (GK1.5), Ly6C Alexa Fluor 647 (HK1.4), CD335 fluorescein isothiocyanate (FITC) (29A1.4) (all from Biolegend); CD11b Alexa Fluor 780 (M1/70, eBioscience); CD41 BUV395 (MWReg30, BD Biosciences). All stains were performed for 12 to 15 min at 4°C. Five milliliters of CountBright counting beads (Invitrogen) were added to each sample such that absolute counts per milliliter of blood could be back calculated. Data were acquired on an LSR Fortessa (BD Biosciences) and analyzed using FlowJo (Tree Star). Prior to sample acquisition, splenocytes were obtained from a healthy mouse spleen, stained with anti-CD4 antibodies, and used for instrument compensation.

### Plasma collection for cross-sectional analyses.

Between three and five infected mice of each strain were euthanized each day from days 3 to 12 postinfection for cross-sectional analysis. Because the WSB/EiJ strain experiences delayed peak infection severity relative to the other mouse strains in this study, three or four WSB/EiJ mice were euthanized each day from days 3 to 17 postinfection for cross-sectional analysis. For each mouse strain, two uninfected control animals were euthanized at baseline and generally on odd-numbered days between days 3 to 12 or days 3 to 17 for WSB/EiJ mice. Euthanasia was performed using carbon dioxide asphyxiation in accordance with Stanford University and APLAC guidelines for humane euthanasia. Following euthanasia, blood was collected via cardiac puncture using 25-gauge (25G) 5/8-in. tuberculin syringes (Fisher Scientific catalog no. 14-841-34). Syringes were primed by filling the syringe barrel with 0.5 M EDTA (pH 8.0) anticoagulant and dispensing all but 50 μl. Collected blood was stored on ice in 1.5-ml Eppendorf tubes for 15 to 45 min before spinning at 1,000 × *g* at 4°C for 5 min in a tabletop centrifuge. Plasma was frozen at −80°C immediately and thawed/refrozen once to aliquot for downstream cytokine, metabolite, and liver enzyme analyses.

### Cytokine analysis.

Seventy-five microliters of plasma was sent to the Human Immune Monitoring Center at Stanford University. Mouse 38-plex kits were purchased from eBiosciences/Affymetrix and used according to the manufacturer’s recommendations with modifications as described below. Briefly, beads were added to a 96-well plate and washed in a Biotek ELx405 washer. Samples were added to the plate containing the mixed antibody-linked beads and incubated at room temperature for 1 h followed by overnight incubation at 4°C with shaking. Cold and room temperature incubation steps were performed on an orbital shaker at 500 to 600 rpm. Following the overnight incubation, plates were washed as described above, and then a biotinylated detection antibody was added for 75 min at room temperature with shaking. The plates were washed as described above, and streptavidin-PE was added. After incubation for 30 min at room temperature, a wash was performed as described above, and reading buffer was added to the wells. Each sample was measured as singletons. The plates were read using a Luminex 200 instrument with a lower bound of 50 beads per sample per cytokine. Custom assay control beads by Radix Biosolutions were added to each well. Samples in which >50% of cytokines returned low bead counts (≤25) were filtered from the data set. Individual data points with a bead count of ≤25 were also removed. For each cytokine, raw median fluorescence intensities are reported as the number of standard deviations from the mean of all samples (Z-scores).

### Metabolite analysis.

One hundred microliters of plasma was shipped to Metabolon (Durham, NC, USA), which performed a combination of gas and liquid chromatography with mass spectrometry (GC/LC-MS). Compounds were identified by comparing sample peaks to an internal Metabolon library of known and unknown compounds. Raw peak values were obtained using area-the-curve. Additional data normalizations were performed by Metabolon to account for sample dilutions and day-to-day variation in instrument performance. The two murine metabolomics experiments in this study (the eight-strain P. chabaudi experiment and the *Ahr^−/−^* P. chabaudi experiment) were measured in two separate Metabolon sample batches.

### Liver enzyme analysis.

In the cross-sectional, eight-mouse strain experiments, 90 μl of plasma was sent to the Veterinary Service Center Diagnostic Lab at Stanford University. Parameters measured were plasma markers of liver damage: aspartate (AST) and alanine (ALT) transaminases. Units are reported as units per liter (U/liter). In follow-up experiments, ALT was measured in 1 μl of plasma collected via cardiac puncture or tail vein bleed using a colorimetric assay (Millipore Sigma catalog no. MAK052) according to the manufacturer’s instructions.

### Comparison of mouse and human metabolic data.

We obtained two human metabolomic data sets to compare with our murine data. The first data set comprised samples from P. falciparum-infected pediatric Malawian patients with signs of cerebral malaria (20). Control samples were obtained from the same patients during the convalescent phase of infection. Metabolic data were obtained from Metabolon, and the scaled imputed ion counts were used for subsequent Z-score transformation and analysis (see below). The second data set is derived from P. falciparum-infected Thai adults in Experiment HuB from the Emory University Malaria Host-Pathogen Interaction Center (MaHPIC) public data release http://www.systemsbiology.emory.edu/research/Public%20Data%20Releases/index.html. Only targeted metabolomics data (Biocrates Life Sciences AG, Innsbruck, Austria) were used for this analysis. Murine data were obtained from Metabolon as described above. To compare among data sets, metabolite names were manually matched, and only metabolites measured in all three data sets (*n* = 42) were included in subsequent analysis. For each metabolite, values were converted to Z-scores by dividing by the mean of values from uninfected C57BL/6 mice (this work), from convalescent individuals (20), or from healthy nonmalarial control patients (MaHPIC). For mouse samples only, we summarized metabolite Z-scores by reporting the median Z-score for each day and mouse strain. For network analysis, we generated a sample-wise Pearson correlation matrix using all Z-scored P. falciparum-infected human samples and median Z-scored mouse samples from acute infection (days 14 to 17 for WSB/EiJ or days 8 to 11 for other strains). In the undirected network, each sample is a node, and edges were drawn between nodes whose correlation coefficients were >0.707 (*R*^2^ > 0.5). We used the same Z-scored metabolite values in the dot plots that show individual metabolites.

### PCA and CCA.

Principal component analysis (PCA) and canonical correlation analysis (CCA) was performed using the phyloseq R package (79) with Bray-Curtis dissimilarity as the distance metric. Infected and uninfected samples from all days and mouse strains were included in the analysis. Scaled imputed ion counts were Z-scored using uninfected C57BL/6 values as the mean, and a pseudocount was added to all values to make the minimum value 1 (Bray-Curtis dissimilarity cannot be computed on negative values). Only metabolites present in ≥80% of samples were included in analysis, and bile acids were removed from analysis prior to correlations because many were inconsistently detected across strains. For CCA for both infections, any metabolite with vector length of >0.1 in CA1 or CA2 was included in the comparison to identify metabolites that overlap between infections.

### Correlation analysis.

Pearson correlations were performed on log_2_-transformed disease severity metrics and log_2_-transformed scaled imputed ion counts. Bile acids were removed from analysis prior to correlations because many were inconsistently detected across strains. A total of 751 metabolites were included in the final correlation analysis. *P* values for correlations were Bonferroni corrected. All samples (infected and uninfected from all days and mouse strains) were included in correlation analysis.

### sPLS-DA.

Partial least-squares discriminant analysis (PLS-DA) is a supervised classification tool that performs dimensionality reduction and variable selection. Sparse PLS-DA (sPLS-DA) is a sparsity-penalized version of PLS-DA that is often applied to “omics” data. We used the mixOmics R package (48) implementation of sPLS-DA to identify metabolites (“features”) that covaried with ALT values, a proxy for liver damage. We first classified samples as low (ALT ≤ 100 U/liter), medium (ALT 101 to 1,000 U/liter), or high (ALT > 1,000 U/liter) liver damage. As in the correlation analysis, scaled, imputed ion counts were log_2_ transformed. Samples from CAST/EiJ and C57BL/6 mice from days 7 to 9 postinfection were included in analysis. Samples from the AhR experiment were from days 7 to 8 postinfection. We prefiltered metabolites to remove those with zero standard deviation in uninfected animals and to remove bile acids because many were inconsistently detected across strains. Component selection (*n* = 3 for both data sets) was chosen to minimize error rates. sPLS-DA is run multiple times on different sample subsets; we selected leave-one-out cross-validation to generate these subsets. Feature stability is the frequency with which a given feature was selected during cross-validation. We allowed for 50 features to be selected for each component to detect as many stable features as possible. For comparison between sPLS-DA and correlations, we included sPLS-DA features with stability frequency of ≥0.9 and correlations with *R*^2^ ≥ 0.5.

### AAV.

Adeno-associated viral (AAV) particles were purchased from Addgene (Watertown, MA, USA). TBG-Cre (AAV.TBG.PI.Cre.rBG, item 107787-AAV8, a gift from James M. Wilson) and TBG-GFP (pAAV.TBG.PI.eGFP.WPRE.bGH, item 105535-AAV8, a gift from James M. Wilson) were thawed immediately prior to use and diluted in sterile, pyrogen-free Dulbecco’s PBS). Virus was injected i.p. 7 days prior to P. chabaudi infection, and monitoring of mouse body weights began 1 week after vector injection. Dosing was based on 53: high = 1 × 10^11^ genome copies (gc), medium = 5 × 10^10^ gc, and low = 1 × 10^10^ gc.

### Dietary restriction.

Infected and uninfected mice were given food and water *ad libitum*. Experimental treatments for uninfected mice were initiated 1 day after infected mice (e.g., day 4 postinfection for infected mice was day 3 postinfection for uninfected mice). Food intake, water consumption, and body weight were measured in all mice starting 2 days before infection. Dietary restriction began at day 4 postinfection, when each uninfected mouse was given only as much food as its respective infected control consumed the previous day. *Ad libitum* water access was maintained for all mice at all times.

### Quantitative amino acid measurements. (i) Analytes and internal standards (ISs).

Arginine (ARG; IS = 13C6, 15N4 ARG), ornithine (ORN; IS = day 7 [d7] ORN), citrulline (CIT; IS = 13C6, 15N4 ARG), asymmetric dimethyl arginine (ADMA; IS = d7 ADMA-HCl), spermidine (SPD; IS = d8 SPD), phenylalanine (PHE; IS = d8, 15N PHE), glutamine (GLUT; IS = d5 GLUT), monosodium glutamate (MSG; IS = d5 GLUT), proline (PRO; IS = d8,15N PHE). Standards were obtained from Cambridge Isotope Laboratories (Tewksbury, MA, USA).

### (ii) Internal standard preparation.

All reference standards were commercially available in powder form. Stock solutions for each standard (10 to 200 mM) were prepared by solubilization in water and diluted further with water-acetonitrile to 200 μM working solutions.

### (iii) Sample preparation.

For plasma amino acid quantitation, 25 μl of frozen plasma was sent for analysis. Five microliters of internal standard solution was added to 20-μl plasma aliquot followed by vortexing. Seventy-five microliters of an ice-cold solution of acetonitrile−0.1% formic acid was added to the sample, followed by vortexing and then centrifugation. Supernatant was transferred to a new vial and analyzed by liquid chromatography coupled to tandem mass spectrometry (LC-MS/MS).

### (iv) Calibration curves.

Individual analyte primary stock solutions were prepared in water (10 to 200 mM). Intermediate stock solution (200 μM) consisting of all unlabeled analytes (ARG, ORN, CIT, ADMA, SPD, PHE, MSG, GLUT, and PRO) was prepared from individual primary stock solutions. This intermediate stock solution was serially diluted with 50× diluted charcoal stripped plasma (CSP) to obtain a series of standard working solutions which were used to generate the calibration curve. Standard working solutions were prepared freshly for sample analysis. Calibration curves were prepared by spiking internal standard solution (10 μM) consisting of six labeled compounds (13C6, 15N4 ARG; d7 ORN; d7 ADMA-HCl; d8 SPD; d8, 15N PHE; d5 GLUT). Because of interference due to endogenous metabolites, calibration curves were prepared in diluted CSP to closely match the study samples. A calibration curve was prepared fresh with each set of samples, and it ranged from 0.2 nM to 4,000 nM.

### (v) Instrumentation.

All analyses were carried out by high-resolution LC-MS/MS using an Agilent Poroshell 120 hydrophilic interaction liquid chromatography (HILIC)-Z column (2.7-μm particle size, 3.0 × 100 mm) on a Quattro Premier triple quadrupole mass spectrometer (Waters) coupled with a 1100 high-performance liquid chromatography (HPLC) system (Agilent). The HPLC conditions were as follows. The column was operated at 30°C at a flow rate of 0.45 ml/min. Mobile phases consisted of solution A (40 mM ammonium formic acid [pH 3.2] in water) and solution B (40 mM ammonium formate [pH 3.2] in 90% acetonitrile). The elution profile was as follows: 90% solution B for 1.5 min, followed by a gradient from 90% to 30% in 8.5 min, then 30 to 95% in 1 min, and held at 90% solution B for a total run time of 16 min. The injection volume was 5 μl.

### (vi) Quantification.

Selected reaction monitoring (SRM) was used for quantification. Analyte mass transitions: arginine: 175.1 → 69.8 (quantifier) and 175.1 → 59.9 (qualifier). Internal standard mass transitions: arginine IS: 185.1 → 74.8 (quantifier) and 185.1 → 63.8 (qualifier); ornithine IS: 139.77 → 76.58 (quantifier) and 139.77 → 98.5 (qualifier). Dwell time was 7 ms for arginine and 10 ms for ornithine. Quantitative analysis was done with QuanLynx software (Waters). Calibration curves were linear (*R* > 0.99) over the concentration range using a weighting factor of 1/*X* where *X* is the concentration. The back-calculated standard concentrations were ±20% from nominal values.

### Arginase activity assay.

Arginase activity assays were performed using a colorimetric assay (Abcam catalog no. 180877) according to the manufacturer’s instructions with the following modifications: hydrogen peroxide standards were not incubated at 37°C; they were prepared immediately prior to addition of reaction mix. Prior to assay, plasma samples (2.5 to 10 μl) were loaded into Amicon 10-kDa spin filters (Fisher Scientific catalog no. UFC501096). Four hundred microliters of water was added, and filters were spun at 15,500 × *g* at 4°C for 10 min in a tabletop centrifuge. After discarding flowthrough, this step was repeated for a total of two spins. Retentate was resuspended in arginase activity buffer (provided by Abcam [catalog no. 180877]) to a final ratio of 2.5-μl original sample to 100-μl total volume. Spin-filtered samples were kept on ice and assayed the same day. To control for interassay variability, arginase activity values were adjusted so that the negative-control sample for each batch (an aliquot from the same uninfected plasma sample) had arginase activity of zero.

### Data availability.

Data for founder strain experiments (survival, disease severity, metabolomics, cytokines, flow cytometry) are provided in Data Set S1 in the supplemental material. Metabolomics data for the founder strains are also available at Metabolomics Workbench, doi:10.21228/M8RX1D, under title “Plasma metabolomics of diverse mouse strains infected with Plasmodium chabaudi.” Data for Fig. 4, C57BL/6 wild type (WT) versus *Ahr^−/−^* data, are from reference 19. Other metabolic data, human and animal, were obtained from existing publications as noted in the text. Data for the remaining figures, and scripts for all figures/tables, are maintained at Github (https://github.com/dave1618/strainsMetabspaper/).

10.1128/mBio.02424-21.8 DATA SET S1 Founder strain malaria experiments. This file contains data from the founder strain experiments, including disease severity, metabolomics and metabolite metadata, and cytokine measurements. It also contains sample metadata, e.g., mouse identifiers and characteristics, and experimental notes. Download Data Set S1, XLSX file, 9.4 MB. Copyright © 2021 Davis et al. 2021 Davis et al. https://creativecommons.org/licenses/by/4.0/ This content is distributed under the terms of the Creative Commons Attribution 4.0 International license.
