# Supplementary material for: Metabolomic Analysis of Diverse Mice Reveals Hepatic Arginase-1 as Source of Plasma Arginase in Plasmodium chabaudi Infection
Source: mBio. 2021 Oct 5;12(5):e02424-21. doi: 10.1128/mBio.02424-21 (PMC8546868; doi:10.1128/mBio.02424-21)
Supplement: TABLE S1 [file mbio.02424-21-st001.docx]

**Table S1. Metabolites whose CCA vectors indicate acute infection in malaria and salmonellosis.** Infected and uninfected samples from all days and mouse strains were included in the analysis. Scaled imputed ion counts were Z-scored using uninfected C57BL/6 values as the mean for malaria data and uninfected DOs as the mean for *Salmonella* data. A pseudocount was added to all values to make the minimum value = 1 (Bray-Curtis dissimilarity cannot be computed on negative values). Only metabolites present in >= 80% of samples were included in analysis, and bile acids were removed from analysis prior to correlations because many were inconsistently detected across strains. For CCA for both infections, any metabolite with vector length > 0.1 in CA1 or CA2 was included in the comparison to identify metabolites that discriminate between health and disease in each infection.

| **#** | **Dataset** | **BIOCHEMICAL** | **HMDB ID** | **CA** | **Length** |
| --- | --- | --- | --- | --- | --- |
| 1 | malaria | N-acetylhomocitrulline |  | 1 | 0.418957 |
| 2 | malaria | N-acetyltyrosine | HMDB00866 | 1 | 0.41193 |
| 3 | malaria | O-sulfo-L-tyrosine |  | 1 | 0.406276 |
| 4 | malaria | 2-methylbutyrylglycine | HMDB00339 | 1 | 0.40272 |
| 5 | malaria | isovalerylglycine | HMDB00678 | 1 | 0.388203 |
| 6 | malaria | 3-(4-hydroxyphenyl)lactate | HMDB00755 | 1 | 0.374664 |
| 7 | malaria | isovalerylcarnitine (C5) | HMDB00688 | 1 | 0.324613 |
| 8 | malaria | 1-methyl-5-imidazoleacetate | HMDB04988 | 1 | 0.322019 |
| 9 | malaria | pipecolate | HMDB00070 | 1 | 0.278304 |
| 10 | malaria | N-acetylphenylalanine | HMDB00512 | 1 | 0.269385 |
| 11 | malaria | 1-carboxyethyltyrosine |  | 1 | 0.258167 |
| 12 | malaria | N-acetyl-1-methylhistidine* |  | 1 | 0.255131 |
| 13 | malaria | N-acetyl-isoputreanine* |  | 1 | 0.249152 |
| 14 | malaria | N('1)-acetylspermidine | HMDB01276 | 1 | 0.244757 |
| 15 | malaria | N-acetylglutamine | HMDB06029 | 1 | 0.238761 |
| 16 | malaria | erucoylcarnitine (C22:1)* |  | 1 | 0.238025 |
| 17 | malaria | 1-methyl-4-imidazoleacetate | HMDB02820 | 1 | 0.235253 |
| 18 | malaria | 2-methylbutyrylcarnitine (C5) | HMDB00378 | 1 | 0.213884 |
| 19 | malaria | isobutyrylcarnitine (C4) | HMDB00736 | 1 | 0.198505 |
| 20 | malaria | N-acetylhistidine | HMDB32055 | 1 | 0.168145 |
| 21 | malaria | beta-hydroxyisovalerate | HMDB00754 | 1 | 0.163105 |
| 22 | malaria | tigloylglycine | HMDB00959 | 1 | 0.161998 |
| 23 | malaria | orotidine | HMDB00788 | 1 | 0.156876 |
| 24 | malaria | N-acetylthreonine | HMDB62557 | 1 | 0.154064 |
| 25 | malaria | phenylacetylcarnitine |  | 1 | 0.151172 |
| 26 | malaria | alpha-hydroxyisovalerate | HMDB00407 | 1 | 0.145217 |
| 27 | malaria | arachidoylcarnitine (C20)* | HMDB06460 | 1 | 0.141756 |
| 28 | malaria | 4-hydroxyphenylacetate sulfate |  | 1 | 0.141059 |
| 29 | malaria | homocitrulline | HMDB00679 | 1 | 0.13628 |
| 30 | malaria | urea | HMDB00294 | 1 | 0.134026 |
| 31 | malaria | lysine | HMDB00182 | 1 | 0.131391 |
| 32 | malaria | octadecadienedioate (C18:2-DC)* |  | 1 | 0.129541 |
| 33 | malaria | eicosenoylcarnitine (C20:1)* |  | 1 | 0.129448 |
| 34 | malaria | 3-hydroxy-2-ethylpropionate | HMDB00396 | 1 | 0.12827 |
| 35 | malaria | 3-methylcrotonylglycine | HMDB00459 | 1 | 0.123872 |
| 36 | malaria | isovalerate (i5:0) | HMDB00718 | 1 | 0.123213 |
| 37 | malaria | tetradecadienoate (14:2)* | HMDB00560 | 1 | 0.122065 |
| 38 | malaria | isocitric lactone |  | 1 | 0.118183 |
| 39 | malaria | 3-hydroxyoleoylcarnitine |  | 1 | 0.117838 |
| 40 | malaria | p-cresol glucuronide* | HMDB11686 | 1 | 0.114862 |
| 41 | malaria | biliverdin | HMDB01008 | 1 | 0.111793 |
| 42 | malaria | isocaproylglycine |  | 1 | 0.110136 |
| 43 | malaria | formiminoglutamate | HMDB00854 | 1 | 0.108469 |
| 44 | malaria | 3-hydroxypalmitoylcarnitine |  | 1 | 0.106013 |
| 45 | malaria | 3-(3-hydroxyphenyl)propionate sulfate |  | 1 | -0.13955 |
| 46 | malaria | N4-acetylcytidine | HMDB05923 | 2 | -0.67247 |
| 47 | malaria | 5,6-dihydrouridine |  | 2 | -0.26831 |
| 48 | malaria | pseudouridine | HMDB00767 | 2 | -0.22272 |
| 49 | malaria | glycerate | HMDB00139 | 2 | -0.21152 |
| 50 | malaria | 3-(3-hydroxyphenyl)propionate sulfate |  | 2 | -0.13703 |
| 51 | malaria | glucuronide of C14H22O4 (1)* |  | 2 | -0.12958 |
| 52 | malaria | orotidine | HMDB00788 | 2 | 0.113632 |
| 53 | *Salmonella* | methylsuccinate | HMDB01844 | 1 | 1.617403 |
| 54 | *Salmonella* | fructose | HMDB00660 | 1 | 0.974378 |
| 55 | *Salmonella* | allantoic acid | HMDB01209 | 1 | 0.70558 |
| 56 | *Salmonella* | uracil | HMDB00300 | 1 | 0.518797 |
| 57 | *Salmonella* | threonate | HMDB00943 | 1 | 0.476231 |
| 58 | *Salmonella* | alpha-tocopherol | HMDB01893 | 1 | 0.455062 |
| 59 | *Salmonella* | oxalate (ethanedioate) | HMDB02329 | 1 | 0.451984 |
| 60 | *Salmonella* | succinate | HMDB00254 | 1 | 0.413888 |
| 61 | *Salmonella* | thymine | HMDB00262 | 1 | 0.321655 |
| 62 | *Salmonella* | glutarate (C5-DC) | HMDB00661 | 1 | 0.314623 |
| 63 | *Salmonella* | tartronate (hydroxymalonate) | HMDB35227 | 1 | 0.302851 |
| 64 | *Salmonella* | gulonate* | HMDB03290 | 1 | 0.275622 |
| 65 | *Salmonella* | propionylcarnitine (C3) | HMDB00824 | 1 | 0.237665 |
| 66 | *Salmonella* | butyrylcarnitine (C4) | HMDB02013 | 1 | 0.237376 |
| 67 | *Salmonella* | isobutyrylcarnitine (C4) | HMDB00736 | 1 | 0.218391 |
| 68 | *Salmonella* | urate | HMDB00289 | 1 | 0.212994 |
| 69 | *Salmonella* | sphinganine-1-phosphate | HMDB01383 | 1 | 0.192435 |
| 70 | *Salmonella* | cytidine | HMDB00089 | 1 | 0.183717 |
| 71 | *Salmonella* | uridine | HMDB00296 | 1 | 0.183102 |
| 72 | *Salmonella* | choline phosphate | HMDB01565 | 1 | 0.166375 |
| 73 | *Salmonella* | valerylglycine | HMDB00927 | 1 | 0.162385 |
| 74 | *Salmonella* | S-1-pyrroline-5-carboxylate | HMDB01301 | 1 | 0.159174 |
| 75 | *Salmonella* | oleoyl-linoleoyl-glycerol (18:1/18:2) [1] | HMDB07219 | 1 | 0.156614 |
| 76 | *Salmonella* | hexanoylcarnitine (C6) | HMDB00705 | 1 | 0.155944 |
| 77 | *Salmonella* | S-methylglutathione |  | 1 | 0.152417 |
| 78 | *Salmonella* | ribulonate/xylulonate* |  | 1 | 0.145635 |
| 79 | *Salmonella* | N-methyl-GABA |  | 1 | 0.140667 |
| 80 | *Salmonella* | gluconate | HMDB00625 | 1 | 0.13926 |
| 81 | *Salmonella* | erythronate* | HMDB00613 | 1 | 0.127001 |
| 82 | *Salmonella* | dimethylglycine | HMDB00092 | 1 | 0.124771 |
| 83 | *Salmonella* | ornithine | HMDB03374 | 1 | 0.121343 |
| 84 | *Salmonella* | decanoylcarnitine (C10) | HMDB00651 | 1 | 0.115574 |
| 85 | *Salmonella* | eicosenoylcarnitine (C20:1)* |  | 1 | 0.114402 |
| 86 | *Salmonella* | glucuronate | HMDB00127 | 1 | 0.109247 |
| 87 | *Salmonella* | N-acetylglutamine | HMDB06029 | 1 | 0.107323 |
| 88 | *Salmonella* | 4-guanidinobutanoate | HMDB03464 | 1 | 0.106669 |
| 89 | *Salmonella* | methylmalonate (MMA) | HMDB00202 | 1 | 0.100256 |
| 90 | *Salmonella* | arginine | HMDB00517 | 1 | -0.18828 |
| 91 | *Salmonella* | 3-phosphoglycerate | HMDB00807 | 1 | -0.17373 |
| 92 | *Salmonella* | ergothioneine | HMDB03045 | 1 | -0.16189 |
| 93 | *Salmonella* | N-acetylglucosaminylasparagine | HMDB00489 | 1 | -0.15824 |
| 94 | *Salmonella* | 2,3-diphosphoglycerate | HMDB01294 | 1 | -0.15441 |
| 95 | *Salmonella* | EDTA | HMDB15109 | 1 | -0.1526 |
| 96 | *Salmonella* | N-acetylneuraminate | HMDB00230 | 1 | -0.15131 |
| 97 | *Salmonella* | S-adenosylhomocysteine (SAH) | HMDB00939 | 1 | -0.1498 |
| 98 | *Salmonella* | adenosine 5'-diphosphoribose (ADP-ribose) | HMDB01178 | 1 | -0.14974 |
| 99 | *Salmonella* | 1,2-dilinoleoyl-GPE (18:2/18:2)* | HMDB09093 | 1 | -0.14662 |
| 100 | *Salmonella* | ophthalmate | HMDB05765 | 1 | -0.14375 |
| 101 | *Salmonella* | N6,N6,N6-trimethyllysine | HMDB01325 | 1 | -0.14355 |
| 102 | *Salmonella* | spermidine | HMDB01257 | 1 | -0.1431 |
| 103 | *Salmonella* | N6-carboxymethyllysine |  | 1 | -0.14305 |
| 104 | *Salmonella* | sphinganine | HMDB00269 | 1 | -0.14228 |
| 105 | *Salmonella* | nicotinamide riboside | HMDB00855 | 1 | -0.14145 |
| 106 | *Salmonella* | malonylcarnitine | HMDB02095 | 1 | -0.141 |
| 107 | *Salmonella* | 1,2-dipalmitoyl-GPE (16:0/16:0)* | HMDB08923 | 1 | -0.14088 |
| 108 | *Salmonella* | nicotinamide | HMDB01406 | 1 | -0.1393 |
| 109 | *Salmonella* | 1-linoleoyl-2-arachidonoyl-GPE (18:2/20:4)* | HMDB09102 | 1 | -0.13875 |
| 110 | *Salmonella* | 1-stearoyl-2-arachidonoyl-GPS (18:0/20:4) | HMDB12383 | 1 | -0.13855 |
| 111 | *Salmonella* | 1-(1-enyl-palmitoyl)-2-oleoyl-GPE (P-16:0/18:1)* | HMDB11342 | 1 | -0.13802 |
| 112 | *Salmonella* | succinylcarnitine (C4-DC) | HMDB61717 | 1 | -0.13761 |
| 113 | *Salmonella* | 14-HDoHE/17-HDoHE |  | 1 | -0.13734 |
| 114 | *Salmonella* | 1-oleoyl-2-linoleoyl-GPE (18:1/18:2)* | HMDB05349 | 1 | -0.13539 |
| 115 | *Salmonella* | adenosine 5'-diphosphate (ADP) | HMDB01341 | 1 | -0.13495 |
| 116 | *Salmonella* | thiamin (Vitamin B1) | HMDB00235 | 1 | -0.13476 |
| 117 | *Salmonella* | 1-palmitoyl-2-stearoyl-GPC (16:0/18:0) | HMDB07970 | 1 | -0.13425 |
| 118 | *Salmonella* | 1-(1-enyl-palmitoyl)-2-arachidonoyl-GPE (P-16:0/20:4)* | HMDB11352 | 1 | -0.13398 |
| 119 | *Salmonella* | undecanedioate (C11-DC) | HMDB00888 | 1 | -0.13362 |
| 120 | *Salmonella* | 1,2-dipalmitoyl-GPC (16:0/16:0) | HMDB00564 | 1 | -0.13357 |
| 121 | *Salmonella* | 1-stearoyl-2-oleoyl-GPE (18:0/18:1) | HMDB08993 | 1 | -0.1323 |
| 122 | *Salmonella* | ceramide (d18:1/20:0, d16:1/22:0, d20:1/18:0)* |  | 1 | -0.13215 |
| 123 | *Salmonella* | 1-palmitoyl-2-oleoyl-GPE (16:0/18:1) | HMDB05320 | 1 | -0.13205 |
| 124 | *Salmonella* | 1-oleoyl-2-arachidonoyl-GPE (18:1/20:4)* | HMDB09069 | 1 | -0.1314 |
| 125 | *Salmonella* | 1-(1-enyl-stearoyl)-2-oleoyl-GPE (P-18:0/18:1) | HMDB11375 | 1 | -0.1313 |
| 126 | *Salmonella* | sphingosine | HMDB00252 | 1 | -0.13071 |
| 127 | *Salmonella* | gamma-glutamylglutamate | HMDB11737 | 1 | -0.13024 |
| 128 | *Salmonella* | 1-oleoyl-2-docosahexaenoyl-GPE (18:1/22:6)* |  | 1 | -0.13006 |
| 129 | *Salmonella* | 2-keto-3-deoxy-gluconate | HMDB01353 | 1 | -0.12941 |
| 130 | *Salmonella* | bacitracin |  | 1 | -0.12885 |
| 131 | *Salmonella* | sphingomyelin (d18:0/18:0, d19:0/17:0)* | HMDB12087 | 1 | -0.12878 |
| 132 | *Salmonella* | biopterin | HMDB00468 | 1 | -0.12848 |
| 133 | *Salmonella* | 3-methyl-2-oxobutyrate | HMDB00019 | 1 | -0.12764 |
| 134 | *Salmonella* | trans-urocanate | HMDB00301 | 1 | -0.12639 |
| 135 | *Salmonella* | 1-(1-enyl-palmitoyl)-2-linoleoyl-GPE (P-16:0/18:2)* | HMDB11343 | 1 | -0.12637 |
| 136 | *Salmonella* | sphingomyelin (d18:0/20:0, d16:0/22:0)* |  | 1 | -0.12559 |
| 137 | *Salmonella* | fructose 1,6-diphosphate/glucose 1,6-diphosphate/myo-inositol diphosphates |  | 1 | -0.12239 |
| 138 | *Salmonella* | behenoyl dihydrosphingomyelin (d18:0/22:0)* | HMDB12091 | 1 | -0.12211 |
| 139 | *Salmonella* | creatine phosphate | HMDB01511 | 1 | -0.1217 |
| 140 | *Salmonella* | p-cresol sulfate | HMDB11635 | 1 | -0.12091 |
| 141 | *Salmonella* | inosine 5'-monophosphate (IMP) | HMDB00175 | 1 | -0.12048 |
| 142 | *Salmonella* | glycerophosphoethanolamine | HMDB00114 | 1 | -0.11993 |
| 143 | *Salmonella* | 12-HETE | HMDB06111 | 1 | -0.11806 |
| 144 | *Salmonella* | 1-palmitoyl-2-arachidonoyl-GPE (16:0/20:4)* | HMDB05323 | 1 | -0.11771 |
| 145 | *Salmonella* | p-hydroxybenzaldehyde | HMDB11718 | 1 | -0.11762 |
| 146 | *Salmonella* | 4-methyl-2-oxopentanoate | HMDB00695 | 1 | -0.11691 |
| 147 | *Salmonella* | thioproline |  | 1 | -0.11662 |
| 148 | *Salmonella* | 1-(1-enyl-stearoyl)-2-arachidonoyl-GPE (P-18:0/20:4)* | HMDB05779 | 1 | -0.11579 |
| 149 | *Salmonella* | cis-urocanate | HMDB34174 | 1 | -0.11503 |
| 150 | *Salmonella* | cysteine | HMDB00574 | 1 | -0.11443 |
| 151 | *Salmonella* | argininosuccinate | HMDB00052 | 1 | -0.11437 |
| 152 | *Salmonella* | 1-(1-enyl-palmitoyl)-2-palmitoyl-GPC (P-16:0/16:0)* | HMDB11206 | 1 | -0.11429 |
| 153 | *Salmonella* | azelate (C9-DC) | HMDB00784 | 1 | -0.11342 |
| 154 | *Salmonella* | N-palmitoyl-sphinganine (d18:0/16:0) | HMDB11760 | 1 | -0.11305 |
| 155 | *Salmonella* | N-formylanthranilic acid | HMDB04089 | 1 | -0.11169 |
| 156 | *Salmonella* | 3-methyl-2-oxovalerate | HMDB03736 | 1 | -0.11149 |
| 157 | *Salmonella* | p-cresol-glucuronide* | HMDB11686 | 1 | -0.10962 |
| 158 | *Salmonella* | N-carbamoylaspartate | HMDB00828 | 1 | -0.10907 |
| 159 | *Salmonella* | N-stearoyl-sphingosine (d18:1/18:0)* | HMDB04950 | 1 | -0.10731 |
| 160 | *Salmonella* | lignoceroyl sphingomyelin (d18:1/24:0) |  | 1 | -0.10727 |
| 161 | *Salmonella* | N-acetylaspartate (NAA) | HMDB00812 | 1 | -0.10538 |
| 162 | *Salmonella* | stearoyl sphingomyelin (d18:1/18:0) | HMDB01348 | 1 | -0.10496 |
| 163 | *Salmonella* | mevalonate | HMDB00227 | 1 | -0.10418 |
| 164 | *Salmonella* | sphingomyelin (d18:1/19:0, d19:1/18:0)* |  | 1 | -0.1027 |
| 165 | *Salmonella* | ribulose/xylulose |  | 1 | -0.10219 |
| 166 | *Salmonella* | glutathione, oxidized (GSSG) | HMDB03337 | 1 | -0.10112 |
| 167 | *Salmonella* | pyridoxamine | HMDB01431 | 1 | -0.10033 |
| 168 | *Salmonella* | methylsuccinate | HMDB01844 | 2 | -1.12784 |
| 169 | *Salmonella* | alpha-tocopherol | HMDB01893 | 2 | -0.40389 |
| 170 | *Salmonella* | daidzein sulfate (1) |  | 2 | -0.2107 |
| 171 | *Salmonella* | phosphoethanolamine | HMDB00224 | 2 | -0.16752 |
| 172 | *Salmonella* | uracil | HMDB00300 | 2 | -0.14789 |
| 173 | *Salmonella* | arginine | HMDB00517 | 2 | -0.12558 |
| 174 | *Salmonella* | daidzein | HMDB03312 | 2 | -0.12077 |
| 175 | *Salmonella* | N-glycolylneuraminate | HMDB00833 | 2 | -0.12033 |
| 176 | *Salmonella* | 1-stearoyl-2-docosahexaenoyl-GPE (18:0/22:6)* | HMDB05334 | 2 | -0.11325 |
| 177 | *Salmonella* | 2-isopropylmalate | HMDB00402 | 2 | -0.10756 |
| 178 | *Salmonella* | 4-hydroxycinnamate sulfate |  | 2 | -0.10385 |
| 179 | *Salmonella* | fructose | HMDB00660 | 2 | 1.416795 |
| 180 | *Salmonella* | allantoic acid | HMDB01209 | 2 | 0.892463 |
| 181 | *Salmonella* | S-1-pyrroline-5-carboxylate | HMDB01301 | 2 | 0.227409 |
| 182 | *Salmonella* | oxalate (ethanedioate) | HMDB02329 | 2 | 0.214903 |
| 183 | *Salmonella* | 1-palmitoyl-GPI (16:0) | HMDB61695 | 2 | 0.20235 |
| 184 | *Salmonella* | threonate | HMDB00943 | 2 | 0.187543 |
| 185 | *Salmonella* | tartronate (hydroxymalonate) | HMDB35227 | 2 | 0.178018 |
| 186 | *Salmonella* | 1-stearoyl-GPI (18:0) | HMDB61696 | 2 | 0.177662 |
| 187 | *Salmonella* | gluconate | HMDB00625 | 2 | 0.173535 |
| 188 | *Salmonella* | butyrylcarnitine (C4) | HMDB02013 | 2 | 0.140661 |
| 189 | *Salmonella* | uridine | HMDB00296 | 2 | 0.135668 |
| 190 | *Salmonella* | ornithine | HMDB03374 | 2 | 0.125774 |
| 191 | *Salmonella* | ribulonate/xylulonate* |  | 2 | 0.114546 |
| 192 | *Salmonella* | hexanoylcarnitine (C6) | HMDB00705 | 2 | 0.111195 |
| 193 | *Salmonella* | oleoyl-linoleoyl-glycerol (18:1/18:2) [1] | HMDB07219 | 2 | 0.111001 |
| 194 | *Salmonella* | stearoyl ethanolamide | HMDB13078 | 2 | 0.105428 |
| 195 | *Salmonella* | succinate | HMDB00254 | 2 | 0.104868 |
