# Supplementary material for: Metabolomic Analysis of Diverse Mice Reveals Hepatic Arginase-1 as Source of Plasma Arginase in Plasmodium chabaudi Infection
Source: mBio. 2021 Oct 5;12(5):e02424-21. doi: 10.1128/mBio.02424-21 (PMC8546868; doi:10.1128/mBio.02424-21)
Supplement: TABLE S2 [file mbio.02424-21-st002.docx]

**Table S2. Student’s t-test results.** T-tests were computed to compare the mean values of metabolites in healthy mice from mouse strains that are resilient (WSB/EiJ, NZO/HILtJ, 129S1/SvImJ, C57BL/6) and non-resilient (PWK/PhJ, A/J, NOD/ShiLtJ, CAST/EiJ) to *Plasmodium chabaudi*. Metabolites that differ significantly (p < 0.05 with Bonferroni correction) between resilient and non-resilient strains are shown.

| **#** | **BIOCHEMICAL** | **HMDB ID** | **T** | **P** | **P (Bonferroni-corrected)** |
| --- | --- | --- | --- | --- | --- |
| 1 | 1-(1-enyl-stearoyl)-2-linoleoyl-GPE (P-18:0/18:2)* | HMDB11376 | 8.704114 | 9.51E-12 | 7.33E-09 |
| 2 | 1-(1-enyl-palmitoyl)-2-linoleoyl-GPC (P-16:0/18:2)* | HMDB11211 | 8.130752 | 1.22E-10 | 9.38E-08 |
| 3 | glucuronide of C14H22O4 (2)* |  | 7.781047 | 3.06E-09 | 2.36E-06 |
| 4 | N-acetyl-3-methylhistidine* |  | -6.60925 | 6.59E-08 | 5.08E-05 |
| 5 | sphingomyelin (d18:1/22:1, d18:2/22:0, d16:1/24:1)* | HMDB12104 | 6.280819 | 9.19E-08 | 7.09E-05 |
| 6 | 1-(1-enyl-palmitoyl)-2-oleoyl-GPC (P-16:0/18:1)* |  | 5.748902 | 3.76E-07 | 0.00029 |
| 7 | betaine | HMDB00043 | -5.75721 | 4.85E-07 | 0.000374 |
| 8 | sphingomyelin (d18:1/18:1, d18:2/18:0) | HMDB12101 | 5.85567 | 5.45E-07 | 0.000421 |
| 9 | oleoyl ethanolamide | HMDB02088 | 5.661693 | 5.64E-07 | 0.000435 |
| 10 | sphingomyelin (d18:2/21:0, d16:2/23:0)* |  | 5.988747 | 7.22E-07 | 0.000557 |
| 11 | linoleoyl-docosahexaenoyl-glycerol (18:2/22:6) [2]* | HMDB07266 | -6.02868 | 1.4E-06 | 0.001081 |
| 12 | homostachydrine* | HMDB33433 | -5.51318 | 1.97E-06 | 0.001518 |
| 13 | 1-palmitoyl-2-gamma-linolenoyl-GPC (16:0/18:3n6)* | HMDB07974 | 5.259053 | 2.32E-06 | 0.001791 |
| 14 | N4-acetylcytidine | HMDB05923 | 5.036803 | 8.8E-06 | 0.006784 |
| 15 | sphingomyelin (d18:2/14:0, d18:1/14:1)* |  | 4.944587 | 1.37E-05 | 0.010538 |
| 16 | sphingomyelin (d18:1/21:0, d17:1/22:0, d16:1/23:0)* |  | 4.964267 | 1.41E-05 | 0.010836 |
| 17 | lysine | HMDB00182 | -4.7763 | 1.86E-05 | 0.014361 |
| 18 | pantothenate | HMDB00210 | -4.72655 | 2.08E-05 | 0.016051 |
| 19 | 1-(1-enyl-stearoyl)-2-arachidonoyl-GPE (P-18:0/20:4)* | HMDB05779 | 4.679796 | 2.21E-05 | 0.017062 |
| 20 | sphingomyelin (d18:1/22:2, d18:2/22:1, d16:1/24:2)* |  | 4.633546 | 2.37E-05 | 0.018245 |
| 21 | 1-(1-enyl-palmitoyl)-2-palmitoyl-GPC (P-16:0/16:0)* | HMDB11206 | 4.635863 | 2.41E-05 | 0.018559 |
| 22 | sphingomyelin (d18:1/25:0, d19:0/24:1, d20:1/23:0, d19:1/24:0)* |  | 4.818136 | 2.52E-05 | 0.019433 |
| 23 | dimethylarginine (SDMA + ADMA) | HMDB01539 | -4.60528 | 2.56E-05 | 0.019736 |
| 24 | 2-methylbutyrylcarnitine (C5) | HMDB00378 | -4.53848 | 3.13E-05 | 0.024109 |
| 25 | behenoyl sphingomyelin (d18:1/22:0)* | HMDB12103 | 4.500664 | 3.85E-05 | 0.029661 |
| 26 | threonine | HMDB00167 | -4.57864 | 5E-05 | 0.038551 |
