# Supplementary material for: Metabolomic Analysis of Diverse Mice Reveals Hepatic Arginase-1 as Source of Plasma Arginase in Plasmodium chabaudi Infection
Source: mBio. 2021 Oct 5;12(5):e02424-21. doi: 10.1128/mBio.02424-21 (PMC8546868; doi:10.1128/mBio.02424-21)
Supplement: TABLE S3 [file mbio.02424-21-st003.docx]

**Table S3.** Metabolites that stably separate CAST from C57BL/6 samples or *AhR^+/+^* C57BL/6 from *AhR^-/-^* C57BL/6 samples on the basis of liver damage using sPLS-DA. “Freq” refers to the frequency with which subsamples of data select a given metabolite during leave-one-out cross-validation (1.00 = 100% of the time). Only metabolites with Freq >= 0.9 are shown.

| **#** | **BIOCHEMICAL** | **Freq** | **HMDB** | **Comparison** |
| --- | --- | --- | --- | --- |
| 1 | adenosine | 1.00 | HMDB00050 | CAST v. B6 |
| 2 | arginine | 1.00 | HMDB00517 | CAST v. B6 |
| 3 | cysteine | 1.00 | HMDB00574 | CAST v. B6 |
| 4 | docosahexaenoylcarnitine (C22:6)* | 1.00 |  | CAST v. B6 |
| 5 | glutamine | 1.00 | HMDB00641 | CAST v. B6 |
| 6 | glycosyl ceramide (d18:1/20:0, d16:1/22:0)* | 1.00 |  | CAST v. B6 |
| 7 | glycosyl ceramide (d18:1/23:1, d17:1/24:1)* | 1.00 |  | CAST v. B6 |
| 8 | glycosyl-N-nervonoyl-sphingosine (d18:1/24:1)* | 1.00 |  | CAST v. B6 |
| 9 | glycosyl-N-palmitoyl-sphingosine (d18:1/16:0) | 1.00 |  | CAST v. B6 |
| 10 | glycosyl-N-stearoyl-sphingosine (d18:1/18:0) | 1.00 |  | CAST v. B6 |
| 11 | glycosyl-N-tetracosadienoyl-sphingosine (d18:1/24:2)* | 1.00 |  | CAST v. B6 |
| 12 | maleate | 1.00 | HMDB00176 | CAST v. B6 |
| 13 | N-acetylmethionine | 1.00 | HMDB11745 | CAST v. B6 |
| 14 | N,N,N-trimethyl-5-aminovalerate | 1.00 |  | CAST v. B6 |
| 15 | N-stearoyl-sphingosine (d18:1/18:0)* | 1.00 | HMDB04950 | CAST v. B6 |
| 16 | N6-methyllysine | 1.00 | HMDB02038 | CAST v. B6 |
| 17 | oleoyl ethanolamide | 1.00 | HMDB02088 | CAST v. B6 |
| 18 | S-methylcysteine | 1.00 | HMDB02108 | CAST v. B6 |
| 19 | S-methylmethionine | 1.00 | HMDB38670 | CAST v. B6 |
| 20 | sphingomyelin (d18:1/20:2, d18:2/20:1, d16:1/22:2)* | 1.00 |  | CAST v. B6 |
| 21 | 1-myristoyl-2-palmitoyl-GPC (14:0/16:0) | 1.00 | HMDB07869 | CAST v. B6 |
| 22 | 1-oleoyl-2-docosahexaenoyl-GPE (18:1/22:6)* | 1.00 |  | CAST v. B6 |
| 23 | 1-oleoyl-2-linoleoyl-GPE (18:1/18:2)* | 1.00 | HMDB05349 | CAST v. B6 |
| 24 | 1-palmitoleoyl-2-linoleoyl-GPC (16:1/18:2)* | 1.00 | HMDB08006 | CAST v. B6 |
| 25 | 1-palmitoyl-2-palmitoleoyl-GPC (16:0/16:1)* | 1.00 | HMDB07969 | CAST v. B6 |
| 26 | 1-stearoyl-2-oleoyl-GPC (18:0/18:1) | 1.00 | HMDB08038 | CAST v. B6 |
| 27 | 2-hydroxybutyrate/2-hydroxyisobutyrate | 1.00 |  | CAST v. B6 |
| 28 | 4-cholesten-3-one | 1.00 | HMDB00921 | CAST v. B6 |
| 29 | formiminoglutamate | 0.97 | HMDB00854 | CAST v. B6 |
| 30 | gamma-glutamylhistidine | 0.97 |  | CAST v. B6 |
| 31 | N-acetylglucosamine/N-acetylgalactosamine | 0.97 | HMDB00215 | CAST v. B6 |
| 32 | palmitoyl sphingomyelin (d18:1/16:0) | 0.97 |  | CAST v. B6 |
| 33 | sphingomyelin (d18:0/18:0, d19:0/17:0)* | 0.97 | HMDB12087 | CAST v. B6 |
| 34 | thymine | 0.97 | HMDB00262 | CAST v. B6 |
| 35 | 1,2-dipalmitoyl-GPC (16:0/16:0) | 0.97 | HMDB00564 | CAST v. B6 |
| 36 | 1-palmitoyl-2-linoleoyl-GPE (16:0/18:2) | 0.97 | HMDB05322 | CAST v. B6 |
| 37 | 5-oxoproline | 0.97 | HMDB00267 | CAST v. B6 |
| 38 | retinal | 0.93 | HMDB01358 | CAST v. B6 |
| 39 | sphingomyelin (d18:2/24:1, d18:1/24:2)* | 0.93 |  | CAST v. B6 |
| 40 | 1-palmitoyl-2-docosahexaenoyl-GPE (16:0/22:6)* | 0.93 | HMDB05324 | CAST v. B6 |
| 41 | 1-palmitoyl-2-oleoyl-GPC (16:0/18:1) | 0.93 | HMDB07972 | CAST v. B6 |
| 42 | arachidonoylcarnitine (C20:4) | 0.90 |  | CAST v. B6 |
| 43 | N-palmitoyl-sphingosine (d18:1/16:0) | 0.90 | HMDB04949 | CAST v. B6 |
| 44 | 4-hydroxyphenylpyruvate | 0.90 | HMDB00707 | CAST v. B6 |
| 45 | arginine | 1.00 | HMDB00517 | AhR WT v. KO |
| 46 | betaine | 1.00 | HMDB00043 | AhR WT v. KO |
| 47 | gamma-glutamylphenylalanine | 1.00 | HMDB00594 | AhR WT v. KO |
| 48 | glycerate | 1.00 | HMDB00139 | AhR WT v. KO |
| 49 | guanidinoacetate | 1.00 | HMDB00128 | AhR WT v. KO |
| 50 | heme | 1.00 | HMDB03178 | AhR WT v. KO |
| 51 | hexadecenedioate (C16:1-DC)* | 1.00 |  | AhR WT v. KO |
| 52 | indoleacetate | 1.00 | HMDB00197 | AhR WT v. KO |
| 53 | indolelactate | 1.00 | HMDB00671 | AhR WT v. KO |
| 54 | linoleoyl ethanolamide | 1.00 | HMDB12252 | AhR WT v. KO |
| 55 | methylsuccinate | 1.00 | HMDB01844 | AhR WT v. KO |
| 56 | N-acetylarginine | 1.00 | HMDB04620 | AhR WT v. KO |
| 57 | N-acetylkynurenine (2) | 1.00 |  | AhR WT v. KO |
| 58 | N-acetylmethionine | 1.00 | HMDB11745 | AhR WT v. KO |
| 59 | N-acetylphenylalanine | 1.00 | HMDB00512 | AhR WT v. KO |
| 60 | N-acetyltryptophan | 1.00 | HMDB13713 | AhR WT v. KO |
| 61 | N-acetyltyrosine | 1.00 | HMDB00866 | AhR WT v. KO |
| 62 | N-formylanthranilic acid | 1.00 | HMDB04089 | AhR WT v. KO |
| 63 | octadecadienedioate (C18:2-DC)* | 1.00 |  | AhR WT v. KO |
| 64 | octadecanedioylcarnitine (C18-DC)* | 1.00 |  | AhR WT v. KO |
| 65 | octadecenedioate (C18:1-DC)* | 1.00 |  | AhR WT v. KO |
| 66 | octadecenedioylcarnitine (C18:1-DC)* | 1.00 |  | AhR WT v. KO |
| 67 | perfluorooctanesulfonate (PFOS) | 1.00 | HMDB59586 | AhR WT v. KO |
| 68 | phenylalanine | 1.00 | HMDB00159 | AhR WT v. KO |
| 69 | phenyllactate (PLA) | 1.00 | HMDB00779 | AhR WT v. KO |
| 70 | riboflavin (Vitamin B2) | 1.00 | HMDB00244 | AhR WT v. KO |
| 71 | S-methylcysteine | 1.00 | HMDB02108 | AhR WT v. KO |
| 72 | sphingosine 1-phosphate | 1.00 | HMDB00277 | AhR WT v. KO |
| 73 | tyrosine | 1.00 | HMDB00158 | AhR WT v. KO |
| 74 | 1,5-anhydroglucitol (1,5-AG) | 1.00 | HMDB02712 | AhR WT v. KO |
| 75 | 1-carboxyethyltyrosine | 1.00 |  | AhR WT v. KO |
| 76 | 1-linoleoyl-2-arachidonoyl-GPE (18:2/20:4)* | 1.00 | HMDB09102 | AhR WT v. KO |
| 77 | 1-oleoyl-2-linoleoyl-GPC (18:1/18:2)* | 1.00 |  | AhR WT v. KO |
| 78 | 1-palmitoyl-GPC (16:0) | 1.00 | HMDB10382 | AhR WT v. KO |
| 79 | 1-palmitoyl-GPE (16:0) | 1.00 | HMDB11503 | AhR WT v. KO |
| 80 | 1-stearoyl-GPC (18:0) | 1.00 | HMDB10384 | AhR WT v. KO |
| 81 | 1-stearoyl-GPE (18:0) | 1.00 | HMDB11130 | AhR WT v. KO |
| 82 | 4-hydroxycinnamate | 1.00 | HMDB02035 | AhR WT v. KO |
| 83 | 5,6-dihydrothymine | 1.00 | HMDB00079 | AhR WT v. KO |
| 84 | 5-dodecenoylcarnitine (C12:1) | 1.00 | HMDB13326 | AhR WT v. KO |
| 85 | xylose | 1.00 | HMDB00098 | AhR WT v. KO |
| 86 | homocitrulline | 0.98 | HMDB00679 | AhR WT v. KO |
| 87 | N-acetylcitrulline | 0.98 | HMDB00856 | AhR WT v. KO |
| 88 | 4-hydroxyphenylpyruvate | 0.95 | HMDB00707 | AhR WT v. KO |
| 89 | glycosyl ceramide (d18:1/20:0, d16:1/22:0)* | 0.90 |  | AhR WT v. KO |
